# Characterization of the immune cell function landscape in head and neck squamous carcinoma to assist in prognosis prediction and immunotherapy

**DOI:** 10.18632/aging.205201

**Published:** 2023-11-10

**Authors:** Wenlun Wang, Zhouyi Zhang, Wenming Li, Dongmin Wei, Jianing Xu, Ye Qian, Shengda Cao, Dapeng Lei

**Affiliations:** 1 Department of Otorhinolaryngology, Qilu Hospital of Shandong University, Jinan, Shandong, P.R. China; 2 NHC Key Laboratory of Otorhinolaryngology, Shandong University, Jinan, Shandong, P.R. China; 3 Key Laboratory for Experimental Teratology of the Ministry of Education and Department of Pathology, School of Basic Medical Sciences, Cheeloo College of Medicine, Shandong University, Jinan, Shandong, P.R. China

**Keywords:** HNSCC, immune cell function, immunotherapy, tumor microenvironment, immunophenotype

## Abstract

Background: The malignant characteristics of cancer depend not only on intrinsic properties of cancer cells but also on the functions of infiltrating immune cells. In this study, we aimed to investigate the functional landscape of immune cells in head and neck squamous cell carcinoma (HNSCC).

Methods: We employed single-sample gene set enrichment analysis to examine the immunophenotypes of HNSCC based on 29 immune cell functions (ICFs) in TCGA and GSE65858 datasets. We analyzed the clinical features, immune microenvironment, molecular profiles, and biological processes. Additionally, we developed and validated an ICF-based risk score for personalized prognosis prediction. We confirmed the value of the ICF score in our cohort using qRT-PCR and immunohistochemistry. Molecular docking was used to predict potential compounds for immunotherapy.

Results: Three immunophenotypes (Immune-L, Immune-M, and Immune-H) were identified in 769 HNSCC samples. The characteristics of Immune-H were consistent with a “Hot” tumor, Immune-L was similar to a “Cold” tumor, and Immune-M exhibited intermediate features. The ICF risk score was associated with immune checkpoints, infiltrating immune cells, tumor mutation burden, and sensitivities to targeted/chemotherapeutic agents. Gene set variation analysis implicated the involvement of metabolic reprogramming pathways in the high-risk group. The combination of “Tumor Immune Dysfunction and Exclusion” and “Immunophenoscore” algorithms indicated that the low-risk group had a higher likelihood of benefiting from immunotherapy. Finally, we identified Eltrombopag and other compounds that may be beneficial for HNSCC immunotherapy.

Conclusion: Our study provides a novel perspective on the tumor microenvironment of HNSCC, aiding in the understanding of HNSCC heterogeneity and the development of personalized/precision medicine.

## INTRODUCTION

Head and neck squamous cell carcinoma (HNSCC) is one of the most aggressive malignant cancers with a high risk of metastasis and mortality [1]. It is estimated that there were approximately 878,348 new cases and 444,347 deaths globally in 2020 [2]. Over the past few decades, the introduction of immune checkpoint inhibitors (ICIs) has revolutionized the treatment approach and prognosis for advanced HNSCC patients [3]. However, the objective response rate to ICIs in recurrent or metastatic HNSCC remains relatively low, ranging from 13% to 18% [4, 5].

Although programmed death ligand-1(PD-L1) has been established as a biomarker for immune checkpoint inhibitors (ICIs) in HNSCC, its limitations have been recognized, as some PD-L1-negative patients still derive benefit from ICIs [6]. Recent studies have proposed that the tumor microenvironment (TME) holds promise as a source of biomarkers for immunotherapy [7, 8]. The malignant phenotype of tumors was influenced not only by inherent characteristics of cancer cells but also by components of the TME [9, 10]. For instance, CD8+ T cells could be present in both “inflamed” and “immune-excluded” tumors [11]. However, in “immune-excluded” tumors, CD8+ T cells were unable to mount an effective immune response against the tumor, potentially due to matrix barriers, T cell exhaustion, and deficiencies in immune factors. Despite HNSCC exhibiting a higher infiltration of immune cells compared to other tumor types, its prognosis remains poor [12]. Hence, further investigations are necessary to unravel the functional status of immune cells in the TME of HNSCC.

Consequently, the objective of this study was to investigate the TME in HNSCC based on the immune cell function, elucidate potential molecular mechanisms, and predict prognosis as well as the responsiveness to immunotherapy in HNSCC.

## MATERIALS AND METHODS

### Data collection

We downloaded transcriptome data (FPKM) and clinical information of 499 HNSCC patients from The Cancer Genome Atlas (TCGA) database. The sequencing platform was Illumina HiSeq. GRCh38 was used for gene annotation.

Transcriptome and clinical data of 270 HNSCC patients were collected from the GEO database (GSE65858). Raw data was extracted using Illumina GenomeStudio, and then log2-transformed and normalized using the Robust Spline Normalization (RSN) method. The annotation platform was GPL10558.

The study was conducted in accordance with the principles outlined in the Declaration of Helsinki and was approved by the Ethics Committee of Qilu Hospital of Shandong University (KYLL-2020(KS)-320). All patients enrolled from our institution provided written informed consent.

### Identification of the immunophenotype based on immune cell function (ICF)

In this study, we employed the immune gene set previously reported by He et al. [13]. This gene set consists of 29 gene signatures associated with immune cell types, functions, and signaling pathways (Supplementary Table 1). To estimate the enrichment scores of these 29 gene signatures in HNSCC samples, we utilized ssGSEA (single sample gene set enrichment analysis). Subsequently, the HNSCC samples were clustered into different immunophenotypes using the “Rtsne” package based on these 29 gene signatures.

The “survival” package was used to conduct Kaplan-Meier survival analysis and log-rank test according to the immunophenotypes.

### Analysis of immune microenvironment characteristics and functional enrichment

The “ESTIMATE” package was employed to evaluate the stromal score, immune score, and tumor purity. Furthermore, the infiltration of 22 immune cells was determined using the “CIBERSORT” package. To compare the expression of immune-related genes among different groups, the “limma”, “ggplot2”, and “ggpubr” packages were utilized, employing the Wilcoxon test. Gene set enrichment analysis (GSEA) was performed using the “enrichplot” and “clusterProfiler” packages to compare the enrichment of functional pathways between different immunophenotypes.

### WGCNA (weighted gene co-expression network analysis)

The “WGCNA” package was used to develop the gene co-expression network. The soft threshold β = 8 was determined as the optimal power value. We set the minimum number of genes in each module to be greater than 60. The gene topology matrix was constructed by using the dynamic cut tree algorithm. The final result was obtained by merging modules with a similarity ≥ 0.75. The correlation between gene modules and clinical traits was determined by the module significance. Genes with weight > 0.3 in the module were screened to develop a protein-protein interaction (PPI) network by using the STRING platform. The hub genes of the PPI network were determined by 12 algorithms from cytoHubba plugin of Cytoscape.

The “clusterProfiler” package was used to conduct GO and KEGG enrichment analysis for module genes, with a significance level of *P* < 0.01.

### Construction and validation of the ICF gene signature

We utilized the “limma” package to analyze the differentially expressed genes (DEGs), and obtained the immune-related gene list from the IMMPORT database. The intersection of the two gene lists yielded immune-related differentially expressed genes (IRDEGs). Next, univariate survival analysis was conducted to select prognosis-related immune genes (PIGs) using the “survival” package.

The “glmnet” package was employed to conduct lasso regression analysis and establish an ICF gene signature. For conducting univariate and multivariate survival analysis, we employed the “survival” package.

### Construction and validation of the ICF score nomogram

We utilized the “surviminer” package to construct the nomogram, and considered factors with *p* < 0.05 as statistically significant. Additionally, we drew ROC and calibration curves to evaluate the performance of the model.

### Analysis of potential transcription factors (TFs) for PIGs

To explore possible TFs for PIGs, we obtained tumor-associated TFs from the Cistrome database (http://www.cistrome.org/). TFs and PIGs with co-expression relationships were selected according to |pearson correlation coefficient| > 0.4 and FDR < 0.01. The correlation of TFs and PIGs was summarized into an alluvial diagram using the “ggalluvial” package. The STRING platform was used to construct the PPI network.

### Integrative analysis of gene set variation analysis (GSVA), tumor mutation burden (TMB), and cancer stem cell index

GSVA is a nonparametric unsupervised clustering method that can analyze specific gene sets in a single sample, thus enabling “pathway” level difference analysis. We utilized the “GSVA” package to evaluate the enrichment of 186 KEGG gene sets. Pathways with *P* < 0.05 and logFC > 0.1 were considered significantly enriched.

We collected mutation information from the TCGA data portal. The “maftool” package was used to investigate comprehensive somatic variants. We employed the chi-square test to compare mutation frequencies across different groups.

Tathiane et al. used machine learning algorithms to analyze the multi-omics features of embryonic stem cells [14]. This method was utilized to calculate the stem cell indexes of TCGA HNSCC. The correlation between mRNA stemness index (mRNAsi) and ICF risk score was evaluated by “limma” package.

### Analysis of treatment sensitivities

The “pRRophetic” package was employed to estimate the IC50 values of 251 chemotherapeutic/targeted agents in HNSCC patients [15]. The Tumor Immune Dysfunction and Exclusion (TIDE) platform was used to calculate T-cell dysfunction and exclusion scores [16]. A higher TIDE score indicated a greater potential for tumor immune escape and might result in poorer immunotherapy efficacy. Pornpimol et al. developed the immunophenoscore (IPS) based on factors influencing tumor immunogenicity [17]. IPS has been proved to be an ideal predictor for ICIs responsiveness. An increased IPS value was indicative of a higher likelihood of benefiting from ICIs.

### Identification of key targets of ICF gene signature

The STRING platform was employed to develop a PPI network of the ICF gene signature with interaction score ≥ 0.4. The Cytoscape software was utilized to determine hub genes according to node connectivity.

### Molecular docking

The molecular structures of 1379 FDA-approved compounds were obtained from the ZINC15 database (https://zinc15.docking.org/). The 3D structure of CD247 was collected from the PDB database (http://rcsb.org/). Firstly, “AutoDockTools-1.5.7” was used to process the structure of the protein, including adding hydrogens, removing the water, and adding the Gasteiger charges. Due to the small molecular weight of CD247 protein, global docking was performed. Small molecules were batch processed by “Obabel” and converted to pdbqt format. Finally, “Vina” was used for batch docking, and the default parameters were adopted.

### *In vitro* validation and survival analyses

Between 2016 and 2017, a total of 80 patients who underwent surgical treatment and were pathologically confirmed to have HNSCC were randomly selected from Qilu Hospital. These patients were followed up until December 2022. Total RNA was extracted from frozen tumor tissues or cultured cells using TRIzol reagent from Absin, China. The cDNA was synthesized using the PrimeScript RT kit from Takara, Japan. RT-PCR was performed using the TB Green Premix Ex Taq II kit, also from Takara, Japan. The primer sequences used are provided in Supplementary Table 2, and GAPDH was used as an internal control. The relative mRNA levels were calculated using the 2−ΔΔCt method.

A paraffin-embedded specimen from each patient was obtained from the pathology department and subjected to immunohistochemical (IHC) staining using an anti-CD247 antibody (1:200, Abcam, UK). The specimens were scored using a semi-quantitative method as previously described [18]. The staining intensity was categorized as follows: 0 (negative), 1 (weak), 2 (moderate), and 3 (strong). Survival analyses were performed using the “survminer” package.

### Cell lines and cell culture

The primary oral mucosal epithelial cells, UMSCC, FaDu, and HN-5 cells were obtained from Meisen Cell Technology Company (China). The cells were maintained in a humidified incubator with 5% CO2 at 37°C while being cultured in DMEM medium (HyClone, USA) supplemented with 10% fetal bovine serum (PAN, Germany), 100 U/mL penicillin, and 100 μg/mL streptomycin. The methods for quantitative real-time PCR have been described above.

## RESULTS

### Identification of the ICF immunophenotype and analysis of the immune microenvironment landscape

**Figure 1:**
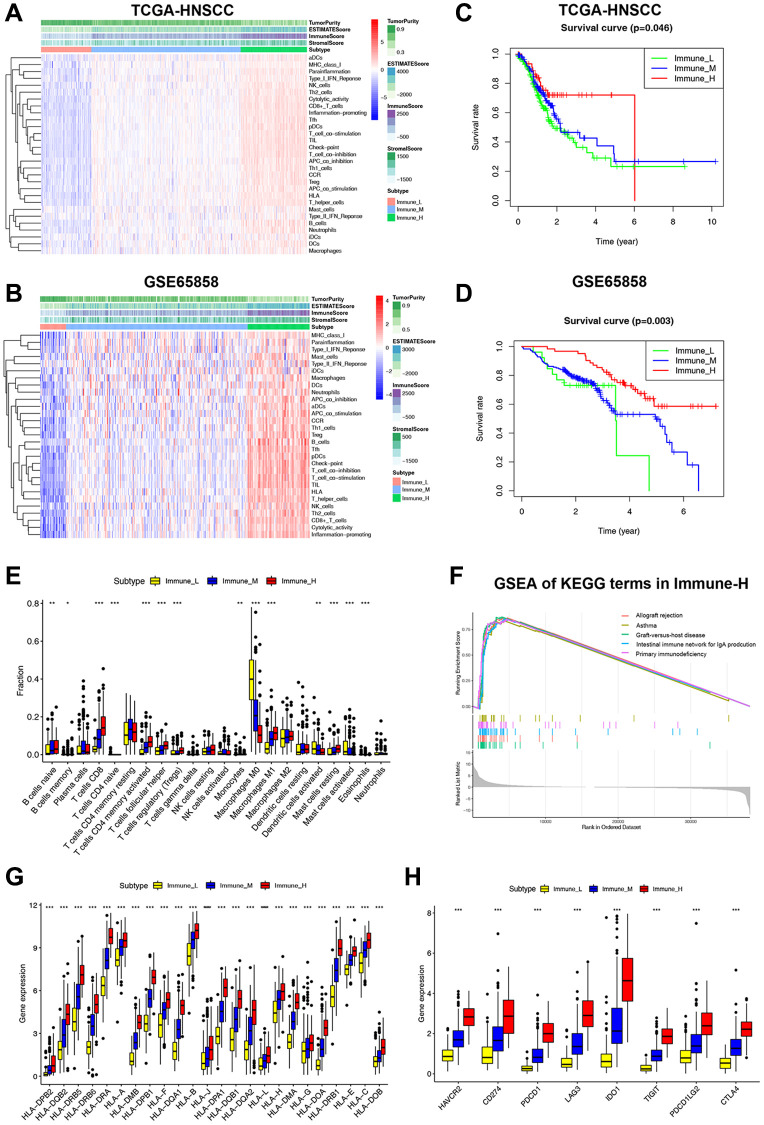
**Classification of HNSCC based on ICF and analysis of the immune microenvironment.** (**A**, **B**) The heatmap shows the enrichment of 29 immune gene signatures and TME components in different ICF subtypes. (**C**, **D**) Kaplan-Meier analysis for overall survival of three ICF subtypes. (**E**) The fractions of immune infiltrating cells in three ICF subtypes. (**F**) GSEA of KEGG pathways in Immune-H. (**G**) The expression levels of HLA alleles in different ICF subtypes. (**H**) The expression levels of immune checkpoints in different ICF subtypes. ^*^p < 0.05, ^**^p < 0.01, ^***^p < 0.001.

We used ssGSEA to assess infiltration levels of 29 functional immune cells in HNSCC samples (Supplementary Table 3). The hierarchical cluster analysis of HNSCC was carried out using T-SNE (t-Distributed Stochastic Neighbor Embedding) algorithm. When divided into three clusters, the intra-cluster consistency and inter-cluster discrimination were found to be the highest (Supplementary Figure 1). The heatmap depicted the diverse degrees of infiltration of 29 functional immune cells across the three clusters, ranging from low to high (Figure 1A). As a result, we have designated the three clusters as follows: “Immune-H” to signify the cluster with high immune cell infiltration, “Immune-M” to represent the cluster with moderate immune cell infiltration, and “Immune-L” to indicate the cluster with low immune cell infiltration. The ESTIMATE results showed that the Immune-H subtype exhibited a higher infiltration of immune and stromal cells compared to the Immune-M and Immune-L subtypes. Correspondingly, the Immune-H subtype had the lowest tumor cell purity. Furthermore, similar immunophenotypes and microenvironment characteristics were also confirmed in GSE65858 (Figure 1C). Survival analysis revealed that the Immune-H subtype had the most favorable survival prognosis of the three subtypes (Figure 1B, 1D).

The CIBERSORT analysis revealed higher fractions of CD8+ T cells, naive B cells, helper follicular T cells, Treg cells, activated CD4+ T cells, M1 macrophages, and resting mast cells in the Immune-H subtype (Figure 1E). On the other hand, the Immune-L subtype showed higher fractions of M0 macrophages, activated dendritic cells, and activated mast cells. The Supplementary Figure 2 and Supplementary Results show the impact of immune cell infiltration on patient survival. GSEA analysis revealed that the Immune-H subtype displayed significant activation of immune rejection and immunodeficiency pathways (Figure 1F). In contrast, except for the chemical carcinogenesis of DNA adducts, no significant enrichment of pathways was observed in the Immune-L subtype (Supplementary Figure 3A, 3B). We also performed WGCNA to identify the immunophenotype-related gene module and hub genes (Supplementary Results and Supplementary Figure 4). Furthermore, it was observed that the expression of HLA alleles increased in correlation with the immune subtypes (Figure 1G). Additionally, the expression of most inhibitory immune checkpoints was found to be elevated in association with the ICF immune subtypes (Figure 1H).

### Construction and validation of the ICF gene signature

**Figure 2:**
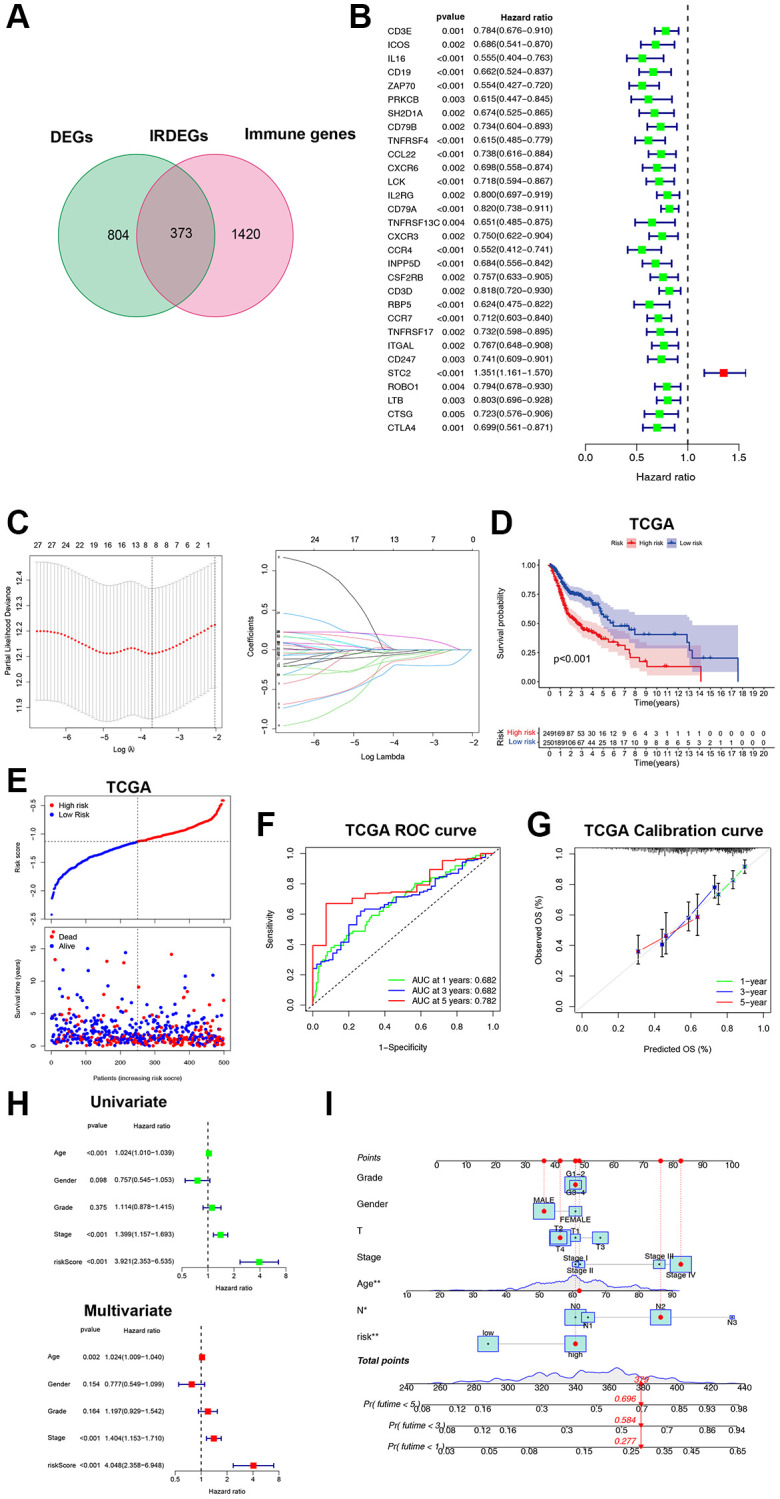
**Construction and validation of the ICF gene signature.** (**A**) Venn diagram shows 373 IRDEGs. (**B**) Univariate Cox analysis for PIGs. (**C**) Lasso regression analysis of PIGs. (**D**) Comparison of overall survival between high- and low-risk groups. (**E**) The correspondence between ICF risk scores and survival outcomes in the training set. The ROC curve (**F**) and calibration curve (**G**) of the ICF score for predicting 1-year, 3-year and 5-year survival. (**H**) Univariate and multivariate survival analyses of ICF score and clinical traits. (**I**) Nomogram based on the ICF score and clinical traits for predicting 1-year, 3-year and 5-year survival. ^*^p < 0.05, ^**^P < 0.01.

It is expensive to determine the ICF immunophenotype by analyzing a large amount of genes through ssGSEA. Consequently, we developed an ICF gene signature for individualized prediction. The TCGA-HNSCC database was used as the training set, and GSE65858 served as the validation set (Supplementary Table 4). We identified 1177 DEGs by comparing the Immune-H and Immune-L subtypes. An intersection of DEGs and immune genes from IMMPORT yielded 373 immune-related DEGs (IRDEGs) (Figure 2A, Supplementary Figure 5A, 5B and Supplementary Table 5). Univariate analysis identified 30 genes associated with prognosis (PIGs, Figure 2B), which were used in LASSO analysis to develop the gene signature: Risk score = CD19 × (−0.0663) + ZAP70 × (−0.1933) + TNFRSF4 × (−0.1303) + CCL22 × (−0.0038) + RBP5 × (−0.0244) + STC2 × (0.1637) + ROBO1 × (−0.0501) + CTSG × (−0.1122) (Figure 2C). We also investigated the possible transcription factors involved in the regulation of PIGs (Supplementary Results and Supplementary Figure 6).

The patients were stratified into high- and low-risk groups based on the median risk score. The survival analysis showed that the low-risk group had a significantly better survival compared to the high-risk group (*P* < 0.01, Figure 2D, 2E). The area under the curve (AUC) values for the ICF gene signature at 1-year, 3-year, and 5-year were 0.682, 0.682, and 0.782, respectively (Figure 2F). These AUC values were higher than those reported by Du et al. for their metabolism-related gene signature (AUC at 1-year, 3-year, and 5-year were 0.66, 0.67, and 0.75, respectively) [19]. The calibration curve demonstrated high accuracy (Figure 2G). Cox regression analyses revealed that age, tumor stage, and the ICF risk score were independent prognostic factors (Figure 2H). The predictive value of the ICF gene signature was further confirmed in the GSE65858 dataset (Supplementary Figure 5C–5F). A nomogram was constructed incorporating the ICF risk score and clinical characteristics to predict 1-year, 3-year, and 5-year survival (Figure 2I). The AUC values for the nomogram were 0.696, 0.735, and 0.709, respectively (Supplementary Figure 5G). The calibration curve indicated a high level of agreement between the predicted and observed outcomes (Supplementary Figure 5H).

**Figure 3:**
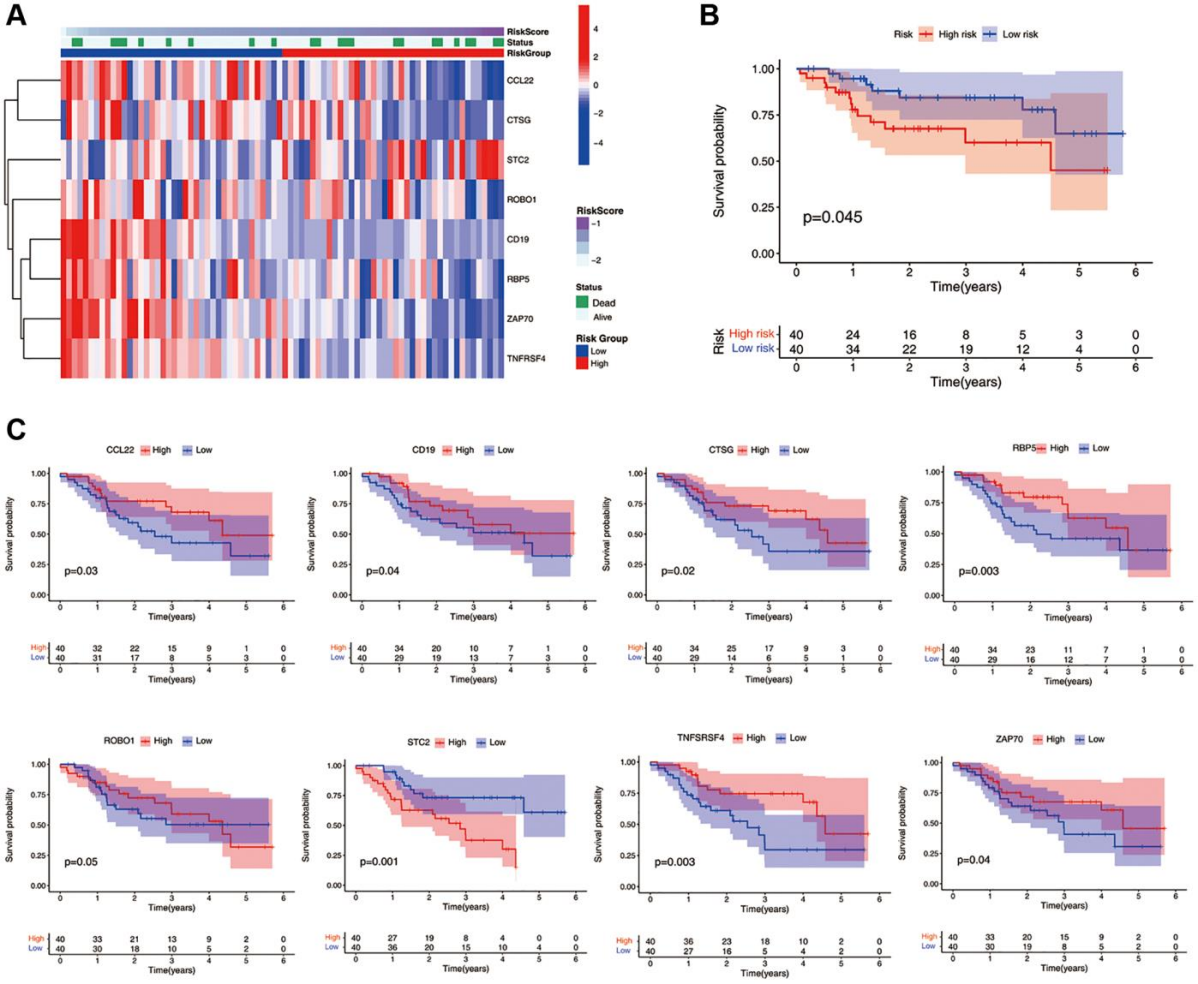
**Validation of the ICF gene signature in Qilu cohort.** (**A**) The heatmap illustrates the expression levels of eight ICF signature genes in the Qilu cohort. (**B**) Kaplan-Meier survival analysis demonstrates the survival outcomes of the high- and low-risk groups in the Qilu cohort. (**C**) Kaplan-Meier survival analysis stratified by the expression levels of eight ICF signature genes in the Qilu cohort.

We collected 80 cases of HNSCC from Qilu Hospital of Shandong University. Gene expression levels were quantified using qRT-PCR. The study cohort was divided into a high-risk group (*n* = 40) and a low-risk group (*n* = 40) based on the median ICF risk score. The heatmap of gene enrichment revealed that STC2 was upregulated in the high-risk group, whereas CCL2, CTSG, ROBO1, CD19, RBP5, ZAP70, and TNFRSF4 were highly expressed in the low-risk group (Figure 3A). Patients in the high-risk group exhibited a significantly lower survival rate compared to those in the low-risk group (*P* = 0.045, Figure 3B). In the stratified survival analysis, STC2 was found to be linked with an unfavorable prognosis in HNSCC patients, whereas CCL2, CTSG, CD19, RBP5, ZAP70, and TNFRSF4 were associated with a favorable prognosis in these patients (Figure 3C).

### Characterization of the ICF score in molecule mechanisms and immune microenvironment

**Figure 4:**
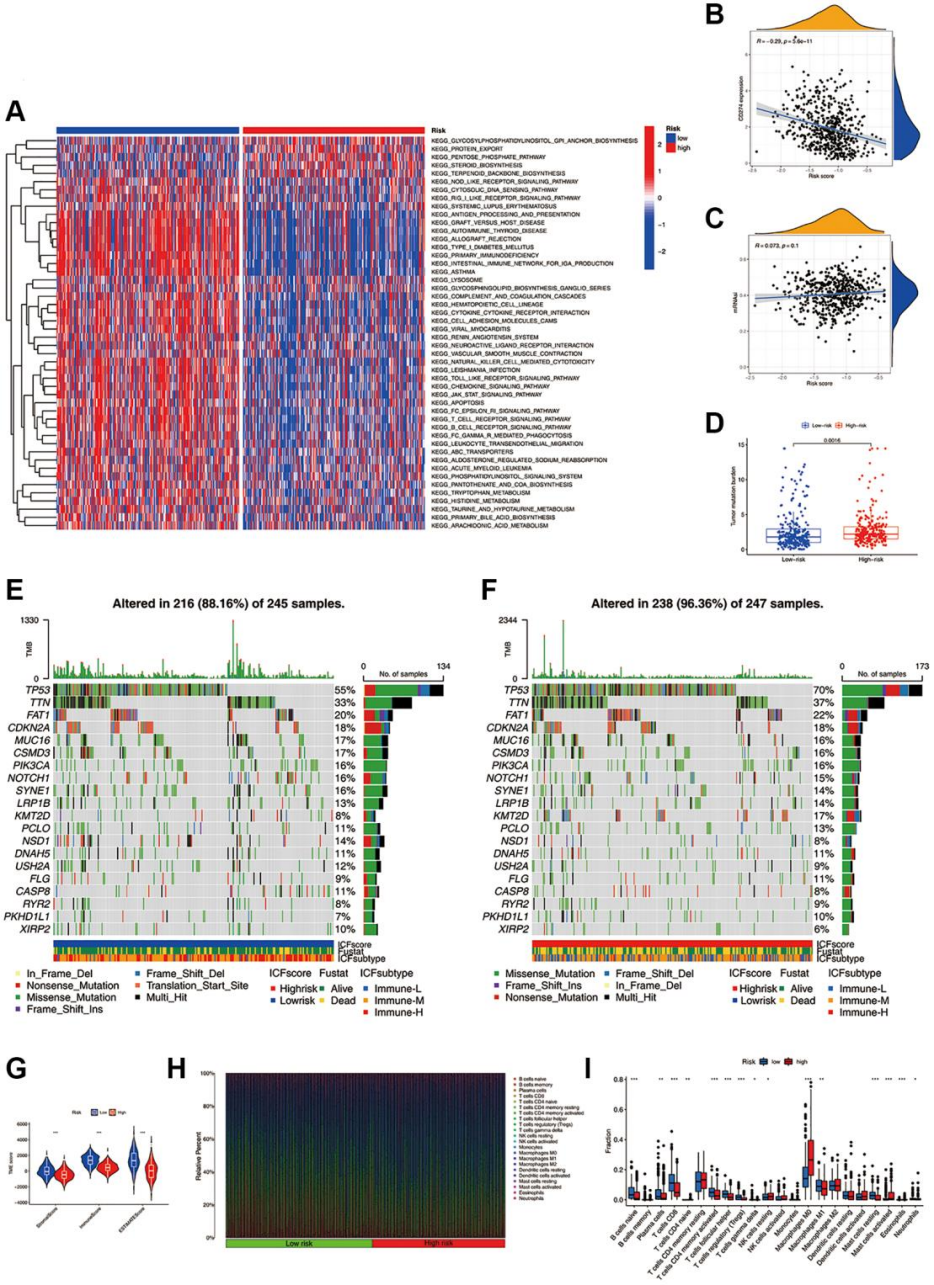
**Molecular mechanisms and the TME landscape of different ICF risk groups.** (**A**) GSVA of KEGG pathways in high- and low-risk groups. (**B**) Correlation of the ICF score and the expression of immune checkpoint CD274 (PD-L1). (**C**) Correlation of the ICF score and cancer stemness indices (mRNAsi). (**D**) Comparison of TMB between high- and low- risk groups. The landscape of somatic variance of the low-risk group (**E**) and high-risk (**F**) group. (**G**) Comparisons of TME components between high- and low-risk groups. (**H**) The bar plot shows the fractions of 22 immune infiltrating cells in high- and low-risk groups. (**I**) Comparisons of the proportions of 22 immune infiltrating cells between high- and low-risk groups.

GSVA was conducted to investigate the molecular mechanisms (Figure 4A). In the high-risk group, pathways such as the biosynthesis of glycosylphosphatidylinositol-anchored proteins (GPI-APs), protein export, and pentose phosphate were significantly enriched. In the low-risk group, pathways of immune activation and immunodeficiency, such as antigen presentation processing and primary immune deficiency, were significantly enriched. The expression of most inhibitory immune checkpoints was found to be negatively correlated with the ICF score (Figure 4B and Supplementary Figure 7). The self-renewal property and therapy resistance of cancer stem cells play a crucial role in the development of malignant tumor phenotypes [14]. However, there was no observed correlation between the ICF score and the cancer stem cell index (Figure 4C). Tumor gene mutation was considered as a prerequisite for immunotherapy. In this study, the high-risk group exhibited a significantly higher TMB compared to the low-risk group (Figure 4D). Survival analysis indicated that the ICF score and TMB had a synergistic effect on prognosis prediction (Supplementary Results and Supplementary Figure 8). Furthermore, somatic variance analysis revealed that the high-risk group had higher frequencies of TP53, KMT2D, and NSD1 mutations compared to the low-risk group (Figure 4E, 4F, and Supplementary Table 6, *p* < 0.05). We have investigated the correlation between the ICF signature genes and the infiltration of immune cells (Supplementary Results and Supplementary Figure 9).

The ESTIMATE algorithm revealed that the high-risk group had lower fractions of immune and stromal components (Figure 4G). According to the CIBERSORT analysis (Figure 4H, 4I), the high-risk group had higher proportions of M0 macrophages, activated mast cells, and resting NK cells. Conversely, the low-risk group had higher fractions of CD8+ T cells, activated CD4+ memory B cells, Treg cells, M1 macrophages, naive B cells, plasma cells, follicular helper T cells, and resting mast cells. We have further compared our ICF immunophenotyping strategy with the previously reported immune typing strategy (Supplementary Results and Supplementary Figure 10).

### Evaluation of the ICF score in predicting chemotherapy and immunotherapy

**Figure 5:**
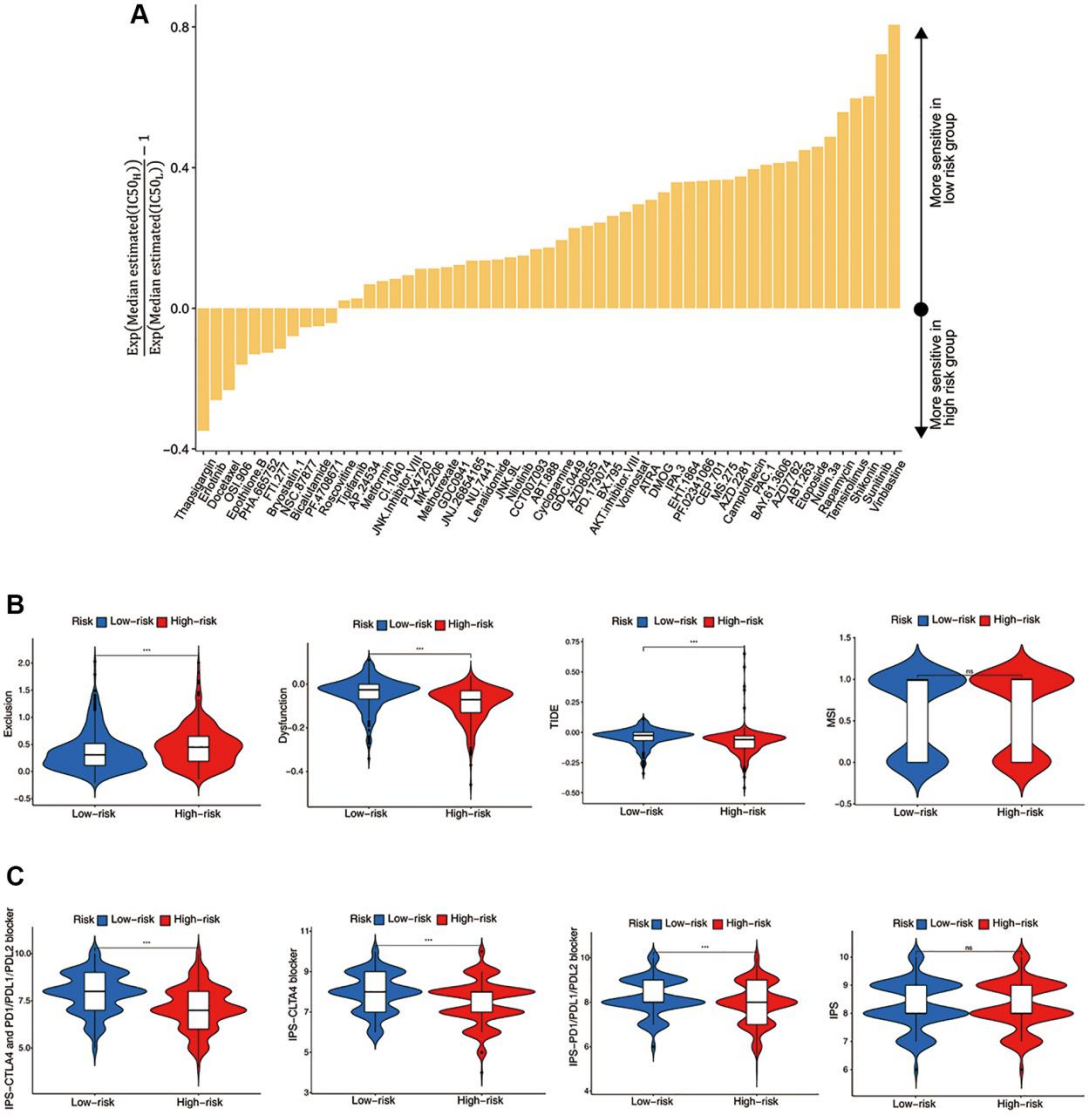
**Evaluation of the ICF score in predicting HNSCC treatment responsiveness.** (**A**) The histogram shows the drugs with significant differences of IC50 value between high- and low- risk groups (Wilcoxon test, *p* < 0.05). (**B**) Comparisons of T cell exclusion and dysfunction scores, the comprehensive TIDE score, and the MSI score between high- and low-risk groups. (**C**) Comparisons of the IPS values between high- and low-risk groups.

The aforementioned evidence indicated that the ICF score was associated with distinct TME characteristics and biological processes, potentially leading to different therapeutic responses. We further investigated the IC50 values of 251 drugs in HNSCC (Figure 5A). Erlotinib and docetaxel were found to be more effective in the high-risk group, while vinblastine, cyclopamine, sunitinib, and rapamycin demonstrated higher sensitivity in the low-risk group. Notably, the sensitivities of commonly used drugs for HNSCC, such as Cisplatin and Bleomycin, did not show significant differences between the high- and low-risk groups (Supplementary Figure 11).

We investigated the utility of the ICF score in predicting immunotherapy response. Figure 5B demonstrated that the low-risk group had significantly lower T-cell exclusion scores, which was consistent with the higher immune components observed in this group. Additionally, the low-risk group exhibited higher T-cell dysfunction scores and comprehensive TIDE scores, which was in line with the activation of immune deficiency and autoimmune pathways in this population. There was no significant difference in microsatellite instability (MSI) between the high-risk and low-risk groups. Finally, the IPS values showed a significant increase in the low-risk group, indicating that this group may be more responsive to ICIs and more likely to benefit from them (Figure 5C).

### Identification of the key target CD247 and prediction of small molecule drugs

**Figure 6:**
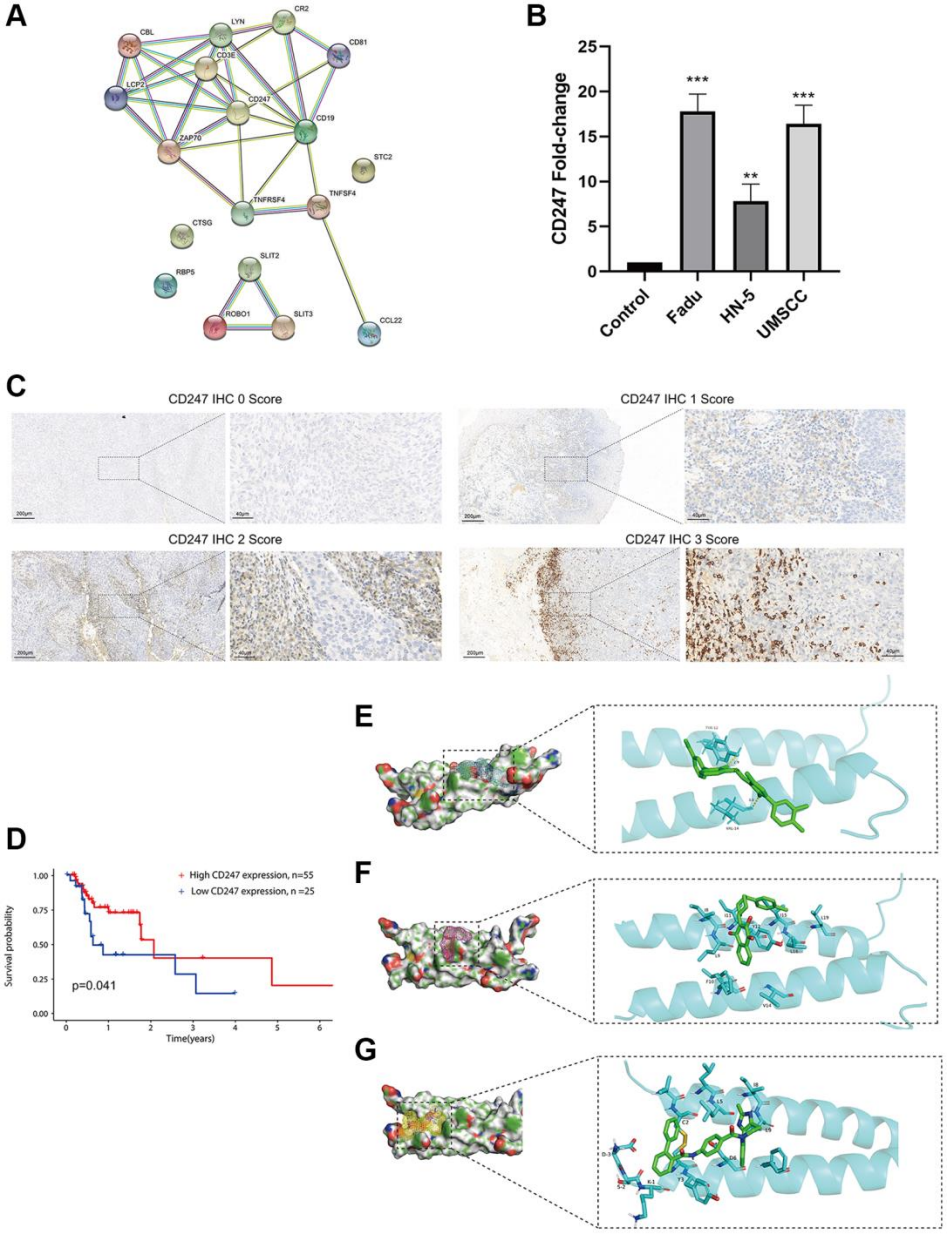
**Identification and validation of the key target and prediction of candidate small molecule compounds.** (**A**) The PPI network of the ICF gene signature (interaction score ≥ 0.4). (**B**) The expression analysis of CD247 in control mucosal epithelia cells and HNSCC cell lines. (**C**) IHC staining of CD247 in HNSCC tissues. (**D**) Kaplan-Meier survival analysis of HNSCC patients according to CD247 IHC scores. The molecular docking diagram shows the interaction between the small molecules Eltrombopag (**E**), ZINC116473771 (**F**), and Conivaptan (**G**) with CD247.

Using the STRING platform, we constructed a protein-protein interaction (PPI) network of the ICF gene signature (interaction score ≥ 0.4, Figure 6A). CD247 was identified as the key target, located at the core of the network. HNSCC cell lines (Fadu, HN-5, and UMSCC) exhibited high expression of CD247 compared to primary oral mucosal epithelial cells (Figure 6B). Immunohistochemical staining was performed on tumor tissues from 80 patients, and the expression of CD247 was scored based on staining intensity (Figure 6C). Our results indicated that patients with high CD247 expression (score 2 and 3) had a more favorable prognosis of survival (Figure 6D), suggesting that CD247 may be a target for tumor immunity in HNSCC and that drugs targeting CD247 could be a potential therapeutic strategy.

In order to identify potential drugs that interact with CD247, we performed molecular docking using over 1300 FDA-approved compounds. Three compounds with the lowest binding energies to CD247 were identified: Eltrombopag (−8.3 kcal/mol, Figure 6E), ZINC116473771 (−8 kcal/mol, Figure 6F), and Conivaptan (−7.8 kcal/mol, Figure 6G). For example, Eltrombopag formed hydrogen bonds with key amino acid residues TYR-12 and VAL-14 in the binding pocket of CD247, indicating a high affinity interaction. The top ten compounds with the highest affinity to CD247 are summarized in Supplementary Table 7. These compounds may be potential immunotherapy drugs for HNSCC.

## DISCUSSION

In the past decade, ICIs represented by anti-PD-L1 antibody have greatly improved the prognosis of advanced HNSCC. Consequently, there has been a growing interest in studying the TME of HNSCC. Zhang et al. proposed an immune classification of HNSCC based on matrix and immune scores, providing valuable insights into the immune microenvironment of HNSCC and its potential implications in immunotherapy [20]. In this study, we identified three immunophenotypes in HNSCC based on 29 functional immune cells. Furthermore, we have utilized a range of methodologies including ssGSEA, WGCNA, GSEA, and GSVA to conduct a comprehensive analysis of the characteristics of the tumor microenvironment (TME). These approaches have allowed us to explore potential molecular mechanisms, establish associations with clinical features, predict prognosis, assess treatment sensitivity, identify key targets, and even predict the efficacy of small molecule drugs.

In our study, the Immune-H subtype exhibited the highest abundance of immune and matrix components within the tumor microenvironment. This subtype was characterized by a significant infiltration of classical anti-tumor immune cells, including CD8+ T cells and M1 macrophages, as well as immunosuppressive cells such as Treg cells. Moreover, both autoimmune and immunodeficiency pathways were prominently enriched in the Immune-H subtype. The expression levels of HLA molecules and ICIs were also notably elevated in the Immune-H subtype. These findings suggested that the Immune-H subtype possessed a high degree of immunogenicity and immune escape potential, similar to that of a “hot” tumor. In contrast, the Immune-L subtype showed a lack of immune cell infiltration and activation of immune-related pathways. This subtype could be considered as a “cold” tumor, where immune cells fail to recognize cancer cells effectively. On the other hand, the Immune-M subtype represented an intermediate status between “hot” and “cold” tumors, suggesting a mixed immune microenvironment.

Goodman et al. performed a retrospective analysis involving 151 patients with multiple types of solid tumors who were treated with ICIs [21]. Their findings revealed that patients with high TMB (TMB-H) exhibited a significantly higher response rate compared to those with low TMB (TMB-L). Several studies have also suggested a positive correlation between TMB-H and improved survival in gastric cancer and ovarian cancer [22, 23]. However, in this study, it was observed that TMB-H was associated with poor survival in patients with HNSCC. Comprehensive analysis suggested that the low-risk group, which mostly comprised patients with TMB-L, may be more favorable for immunotherapy. This group exhibited increased immune cell infiltration, immune checkpoint expression, HLA molecule expression, and higher IPS values, suggesting a stronger immunogenicity. Consequently, the effectiveness of ICIs treatment in HNSCC may be influenced by multiple factors due to the heterogeneity of the tumor microenvironment.

M0 macrophages were typically considered as the precursor cells that can differentiate into M1 or M2 macrophages, but they did not exhibit specific functional properties themselves [24]. In our study, we observed a high infiltration of M0 macrophages in the high-risk group, implying their tumor-promoting role. Recent evidence has also revealed the extensive differentiation and tumorigenic potential of M0 macrophages in hepatocellular carcinoma and glioma, challenging the traditional M1/M2 paradigm [25, 26]. Chan et al. reported that upon contact with cancer cells, active NK cells could transform into a resting state, and played a role in tumorgenesis and metastasis [27]. This was in line with our study, resting NK cells had a higher proportion in the high-risk group. Our results revealed the activation of multiple metabolic reprogramming pathways in the high-risk group, including steroid biosynthesis and the pentose phosphate pathway. Similarly, Ringel et al. demonstrated that tumor cells upregulate the free fatty acid pathway to competitively suppress the fuel utilization and functionality of CD8+ T cells, thereby promoting tumor development [28].

In order to identify potential targets for immunotherapy, we focused on the hub gene CD247 of the ICF gene signature. Down-regulation of CD247 has been observed to induce immunosuppression in chronic inflammation and tumors [29]. To explore potential interactions with CD247, we screened over 1300 small molecule compounds approved by the FDA. Eltrombopag, a thrombopoietin receptor agonist that stimulates the proliferation of megakaryocytes [30], may be a candidate small molecule for immunotherapy if it can also act as a TCR agonist to promote anti-tumor immunity. However, further experiments are needed to verify this speculation.

Our study has a few limitations that should be acknowledged. Firstly, the ICF-based classification strategy requires validation in larger multicenter cohorts. Secondly, due to the absence of expression data from HNSCC patients undergoing immunotherapy, our findings need to be further confirmed in HNSCC patients who have received immunotherapy.

## CONCLUSIONS

Based on ICF, we have classified HNSCC into three distinct immunophenotypes (Immune-L, Immune-M and Immune-H), which were associated with different clinical features, molecular profiles, and biological processes. The characteristics of Immune-H were consistent with those of “Hot” tumors, while Immune-L was in line with “Cold” tumors, and Immune-M was intermediate between the two. Furthermore, the ICF score, which was constructed based on the immunophenotypes, demonstrated robust performance in predicting prognosis. The ICF score was also found to be associated with TMB, immune checkpoints, HLA alleles, and responsiveness to chemotherapy and immunotherapy. Collectively, our work provides a new perspective on the immune microenvironment of HNSCC, which facilitates the interpretation of the HNSCC heterogeneity and developing personalized/precision medicine.

## Supplementary Materials

Supplementary Results

Supplementary Figures

Supplementary Tables 2, 4 and 6-7

Supplementary Tables 1, 3, 5 and 8
