# Supplementary material for: Characterization of the immune cell function landscape in head and neck squamous carcinoma to assist in prognosis prediction and immunotherapy
Source: Aging (Albany NY). 2023 Nov 10;15(21):12588–617. doi: 10.18632/aging.205201 (PMC10683602; doi:10.18632/aging.205201)
Supplement: Supplementary Results [file aging-15-205201-s001.pdf]

SUPPLEMENTARY RESULTS

Impact of immune cell infiltration on patient survival

Survival analysis was performed to assess the impact of immune cell infiltration on patient outcomes (Supplementary Figure 2). The results revealed that patients with higher levels of infiltration of naive B cells, resting dendritic cells, resting mast cells, plasma cells, helper follicular T cells, and Treg cells had more favorable prognoses. These immune cell types were predominantly abundant in the Immune-H subtype. On the other hand, patients with higher levels of activated mast cells, neutrophils, resting CD4+ memory cells, and M2 macrophages had poorer prognoses.

Identification of the immunophenotype-related gene module and hub gene

WGCNA analysis identified 16 gene modules potentially responsible for the immunophenotype (Supplementary Figure 4A). The black module consisted of 1907 genes (Supplementary Table 8) that exhibited a positive correlation with the Immune-H subtype and a negative correlation with the Immune-L subtype (Supplementary Figure 4B). Moreover, the black module gene showed a significant association with survival time. GO and KEGG analyses indicated that black module genes were significantly enriched in pathways related to immune cell communication (Supplementary Figure 4C, 4D). A protein-protein interaction (PPI) network was constructed, consisting of 154 hub genes from the black module (Supplementary Figure 4E). Within this network, SAHS3, CD53, and NCKAP1L occupied central positions. Additionally, SASH3, CD53, and NCKAP1L showed higher expression levels in the Immune-H subtype, while lower expression levels in the Immune-L subtype (Supplementary Figure 4F). These genes might possess the strongest biological significance within the black module.

Exploration of transcription factors in the regulation of prognosis-related immune genes (PIGs)

We also investigated the involvement of transcription factors in the regulation of PIGs through co-expression analysis (Supplementary Figure 6A). Our analysis revealed fifteen transcription factors, including FOXP3, IRF1, and STAT4, which exhibited redundancy in the regulation of PIGs. These transcription factors may play crucial roles in driving different risk groups. Additionally, the PPI network displayed potential interactions between PIGs and the identified transcription factors. Notably, CTLA4, CD3D, and CD19 were found to be positioned at the core of the network (Supplementary Figure 6B).

Synergistic effect of the ICF Score and TMB on prognosis

Survival analysis revealed that patients with TMB-H had significantly worse survival compared to those with TMB-L (Supplementary Figure 8A). Furthermore, the combination of the ICF score and TMB had a synergistic effect in predicting prognosis. Patients with both TMB-H and high-risk scores had the shortest median survival time, while those with TMB-L and low-risk scores had the most favorable survival outcome (Supplementary Figure 8B).

Correlation between ICF signature genes and infiltration of immune cells

We investigated the correlation between the expression levels of the ICF signature genes and the infiltration of immune cells (Supplementary Figure 9). The majority of the signature genes displayed significant correlations with immune cell infiltration. Notably, ZAP70 exhibited a positive correlation with CD8+ T cells and follicular helper T cells, while showing a negative correlation with activated mast cells and M0 macrophages. Besides, CD19 demonstrated a positive correlation with plasma cells and naive B cells, but a negative correlation with M0 macrophages.

Advantages of ICF immunophenotyping strategy in HNSCC classification

We further compared our ICF immunophenotyping strategy with a previously reported immune typing strategy by Ve´ steinn et al. In their study, solid tumors were categorized into six immune subtypes, with HNSCC primarily classified as C1 (wound healing) and C2 (INF-γ dominant) [1]. However, this classification system heavily relied on the differentiation of CD4+ T cells, which may not effectively distinguish HNSCC (Supplementary Figure 10). In contrast, our method divided HNSCC into three ICF subtypes and further stratified them into high- and low-risk groups. The Immune-H subtype predominantly fell into the low-risk group, while the Immune-L subtype mainly belonged to the high-risk group. Additionally, the low-risk group showed a higher proportion of patients with improved survival outcomes. As a result, our ICF-guided immunophenotyping strategy demonstrated clear advantages in characterizing HNSCC.

Supplementary References

1. Thorsson V, Gibbs DL, Brown SD, Wolf D, Bortone DS, Ou Yang TH, Porta-Pardo E, Gao GF, Plaisier CL, Eddy JA, Ziv E, Culhane AC, Paull EO, and Cancer Genome Atlas Research Network. The Immune Landscape of Cancer. Immunity. 2018; 48:812–30.e14. https://doi.org/10.1016/j.immuni.2018.03.023 PMID:29628290

Supplementary Figures

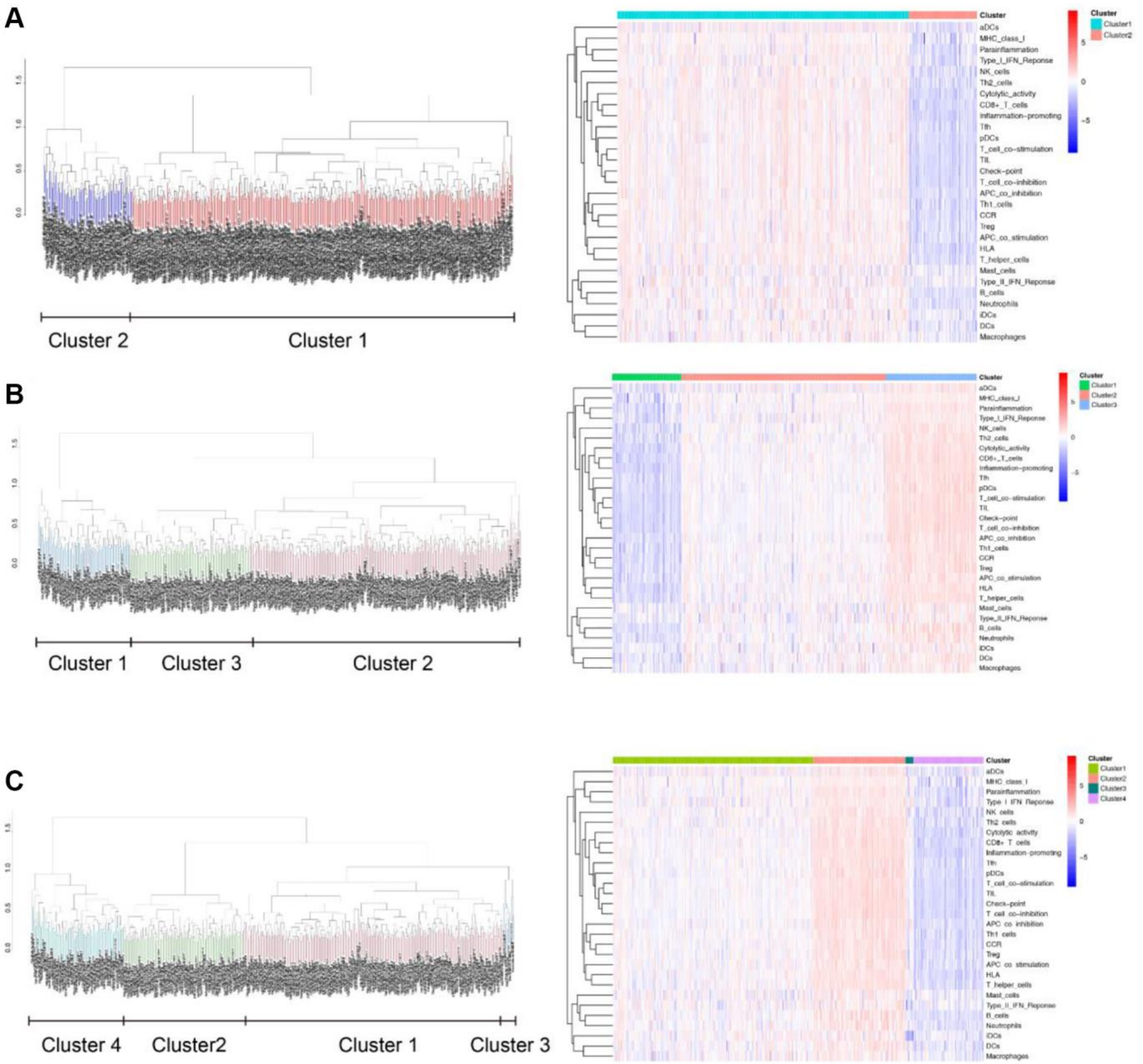

Supplementary Figure 1. Cluster dendrogram (left panel) and the enrichment analysis of 29 functional immune cells (right panel) when TCGA-HNSCC samples were divided into 2 (**A**), 3 (**B**) and 4 (**C**) clusters.

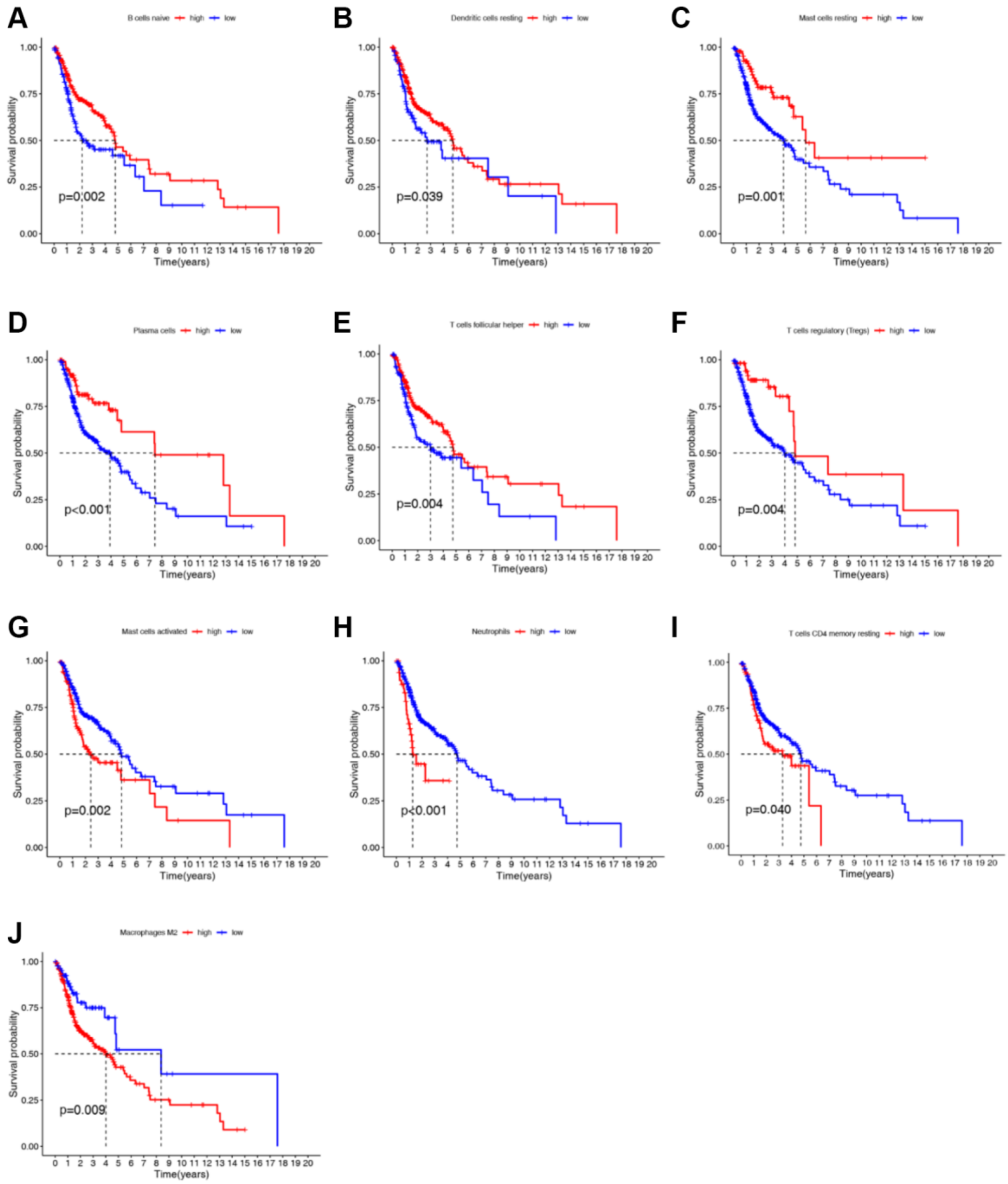

Supplementary Figure 2. Survival analyses based on immune cell infiltration. (**A**) naïve B cells, (**B**) resting dendritic cells, (**C**) resting mast cells, (**D**) Plasma cells, (**E**) T follicular helper cells, (**F**) regulator T cells, (**G**) activated mast cells, (**H**) Neutrophils, (**I**) memory resting CD4+ T cells, and (**J**) M2 macrophages.

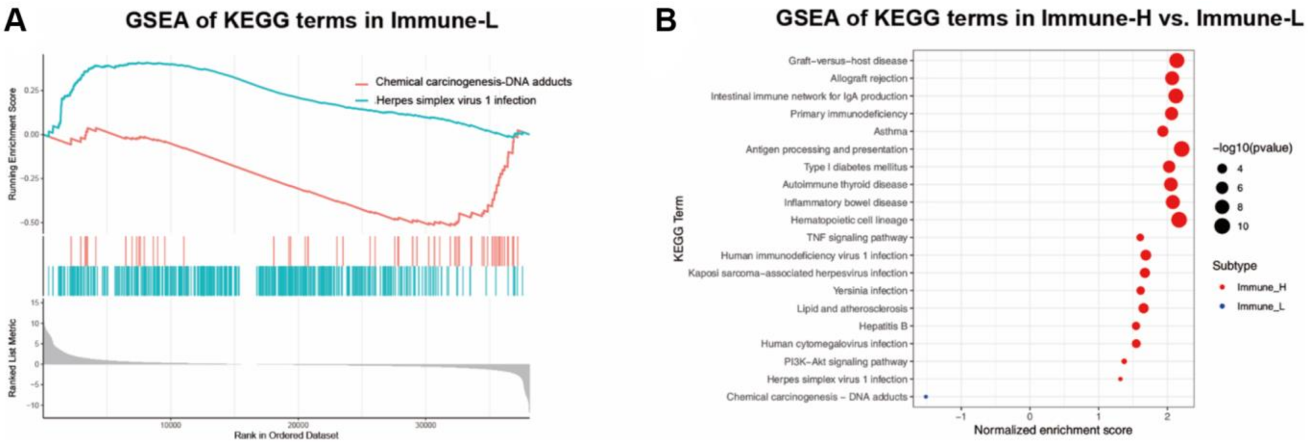

Supplementary Figure 3 Biological processes in different immunophenotypes. (**A**) GSEA shows only the chemical carcinogenesis (DNA adducts) pathway is enriched in the Immune-L subtype. (**B**) The bubble plot shows the enrichment result of KEGG terms in Immune-H and Immune-L subtypes.

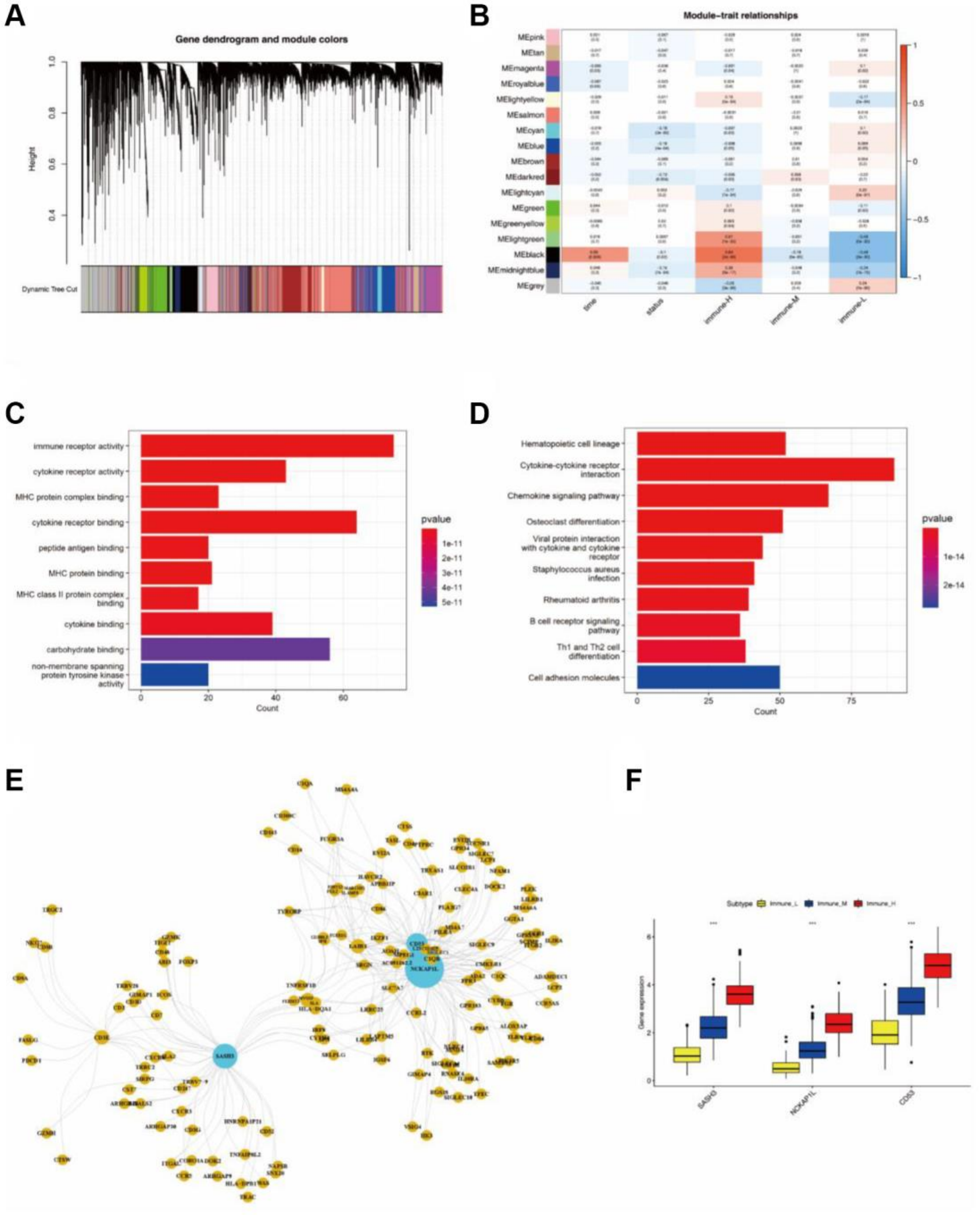

Supplementary Figure 4. Identification of the immunophenotype-related gene module by WGCNA. (**A**) Cluster dendrogram of gene co-expression modules after merged (1-TOM). (**B**) Correlation analysis of gene modules and phenotypes of HNSCC. GO functional enrichment (**C**) and KEGG pathway enrichment (**D**) analyses for genes in the black module of WGCNA. (**E**) The protein-protein interaction network of the hub genes in the black module. (**F**) The expression levels of SAHS3, CD53 and NCKAP1L in the three immunophenotypes.

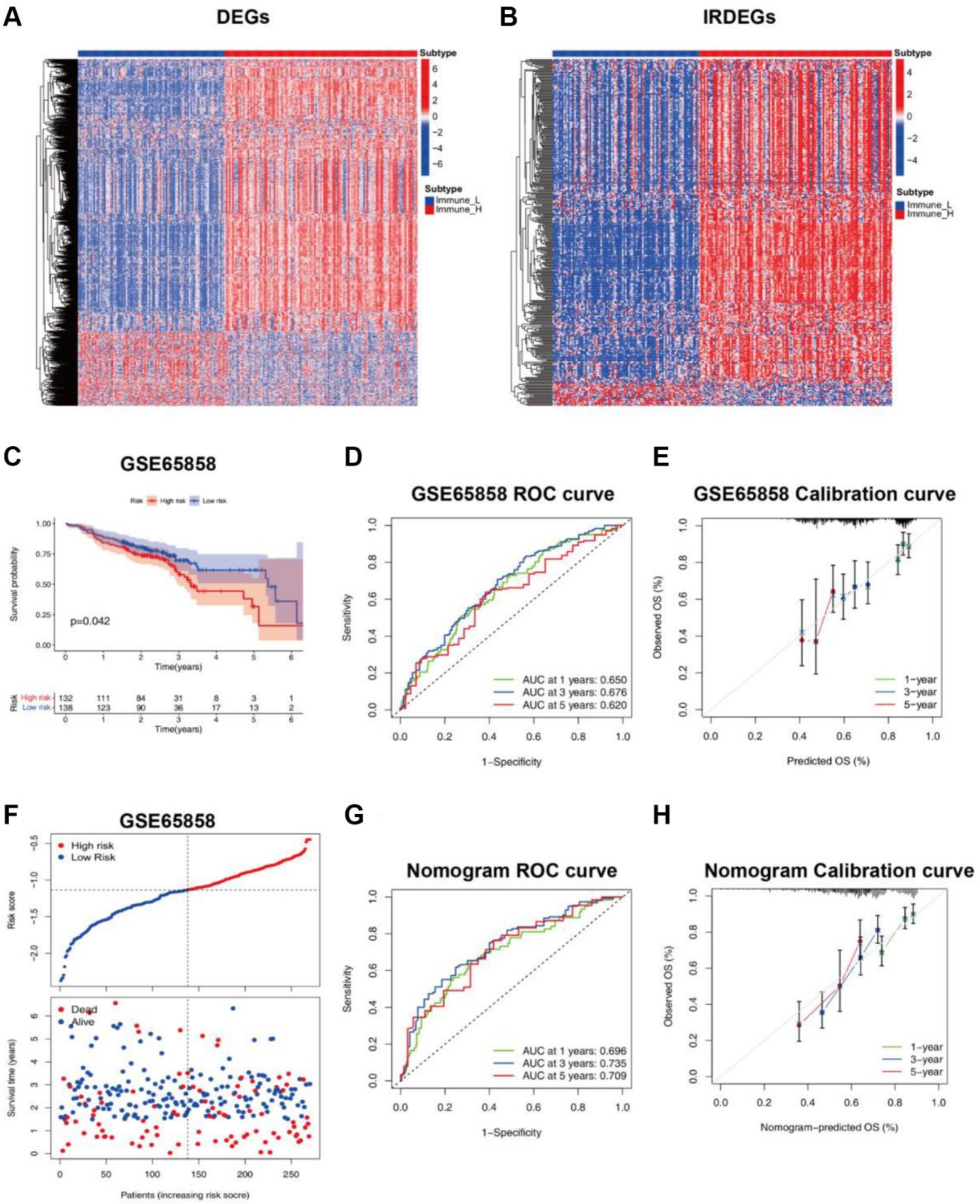

Supplementary Figure 5. Construction and validation of the ICF gene signature. Heatmap of DEGs (**A**) and IRDEGs (**B**) between the Immune-H and Immune-L subtypes. (**C**) Comparisons of overall survival between high- and low-risk groups in the test set. The ROC curve (**D**) and calibration curve (**E**) of the ICF score for predicting 1-year, 3-year and 5-year survival in the test set. (**F**) Correspondence between risk scores and survival in the test set. The ROC curve (**G**) and calibration curve (**H**) of the nomogram for predicting overall survival at 1-year, 3-year and 5-year.

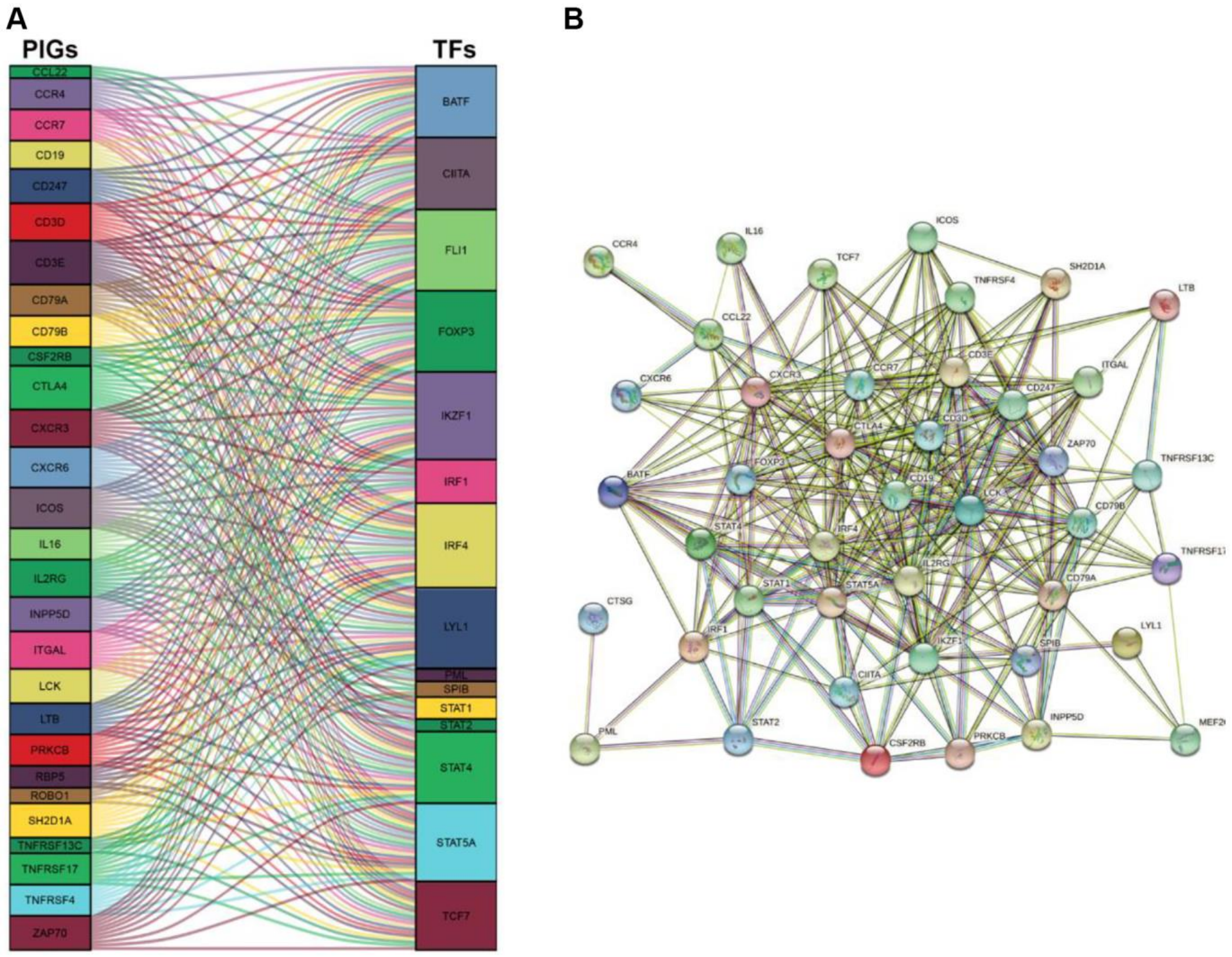

Supplementary Figure 6. Analysis of candidate transcription factors (TFs) for prognosis-related immune genes (PIGs). (**A**) The alluvial diagram shows the TFs co-expressed with PIGs, and the lines indicate the co-expressed relationship between the two. (**B**) Protein-protein interaction network of PIGs and TFs.

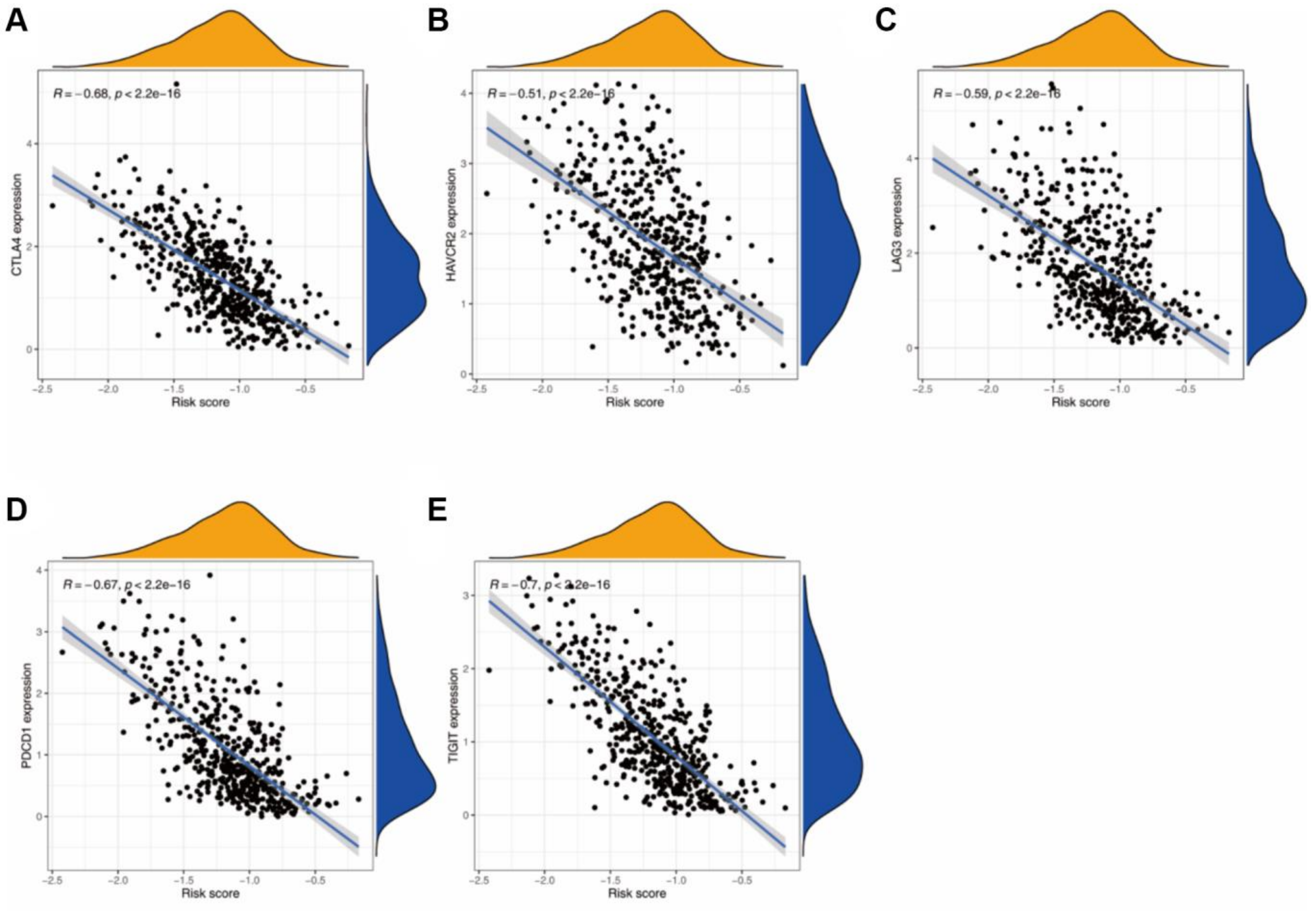

Supplementary Figure 7. The correlations between immune checkpoints and ICF scores in HNSCC. CTLA4 (**A**), HAVCR2 (**B**), LAG3 (**C**), PDCD1 (**D**), and TIGIT (**E**).

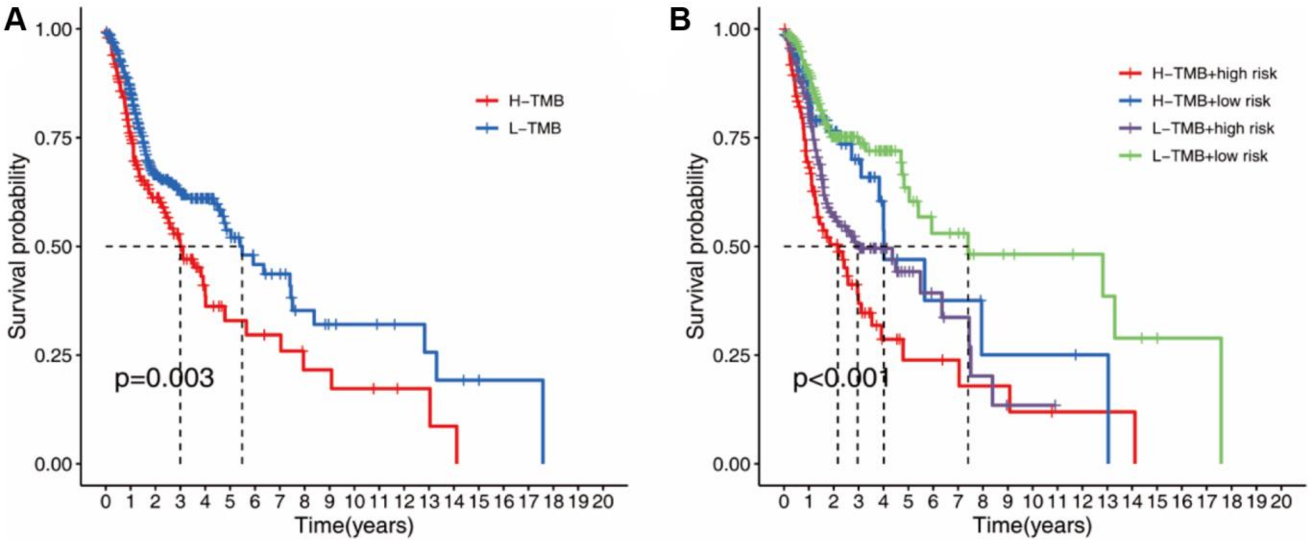

Supplementary Figure 8. Survival analysis for HNSCC patients stratified by TMB and ICF risk score. (**A**) Kaplan-Meier survival analysis for HNSCC patients with different TMB. (**B**) Kaplan-Meier survival analysis for patients according to TMB and ICF score stratifications.

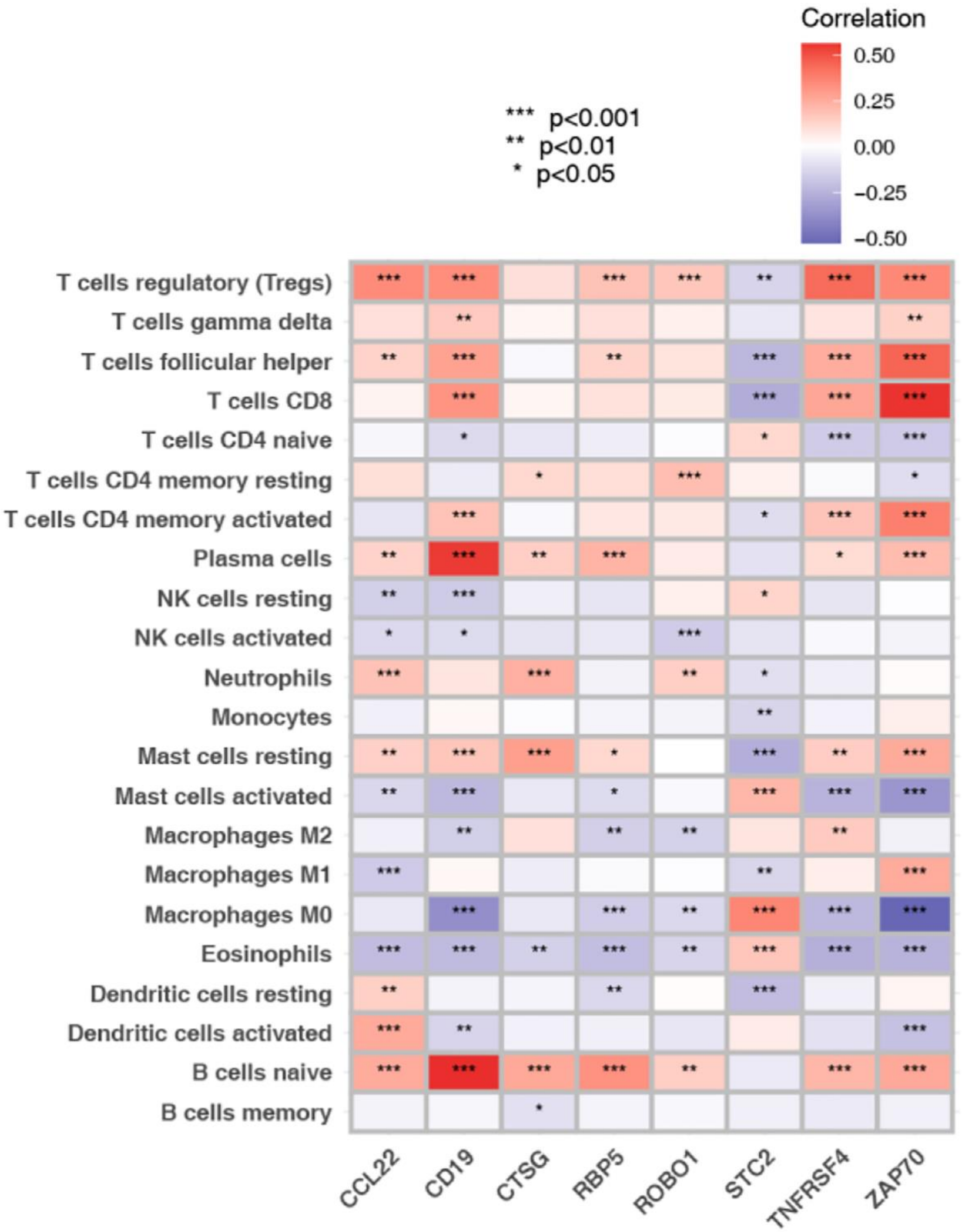

Supplementary Figure 9. Correlation analyses between the model genes and immune infiltrating cells.

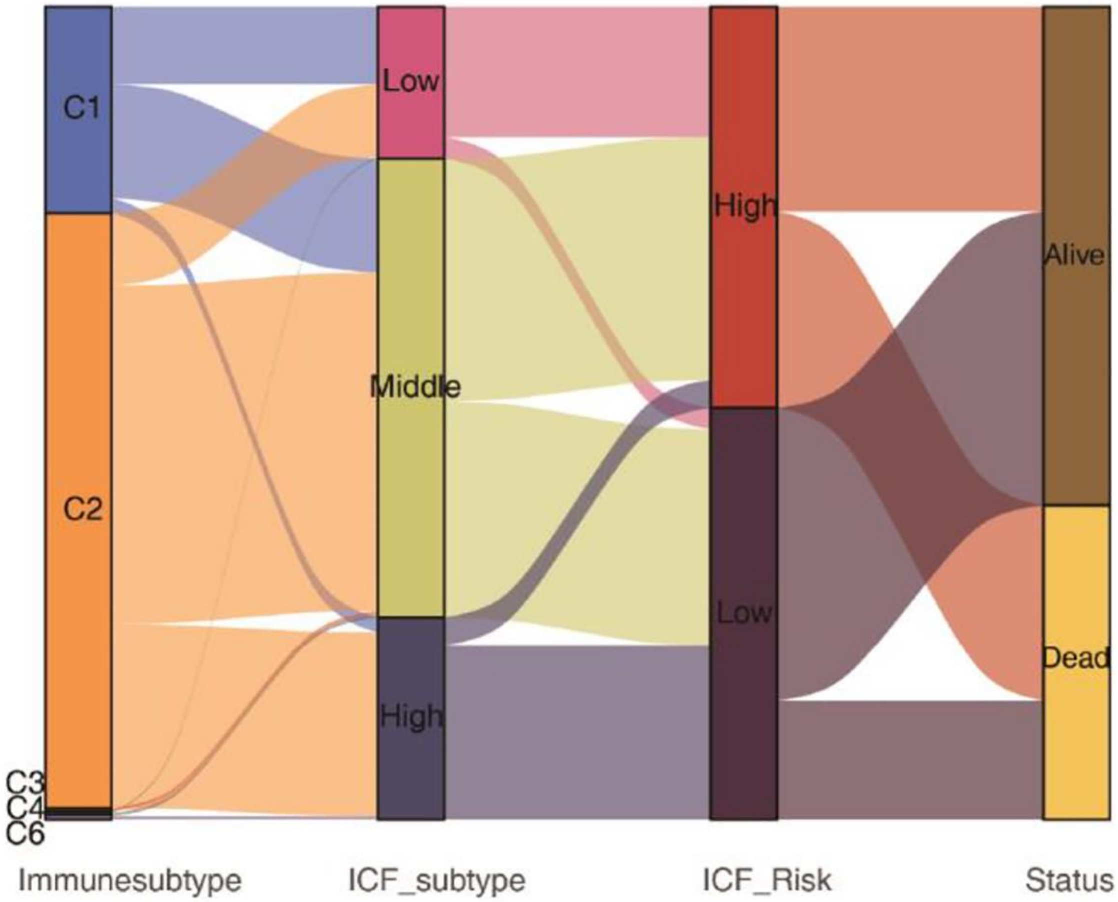

Supplementary Figure 10. The alluvial diagram shows the correspondence of pan-cancer immune subtypes, ICF subtypes, ICF risk groups and the survival status in HNSCC.

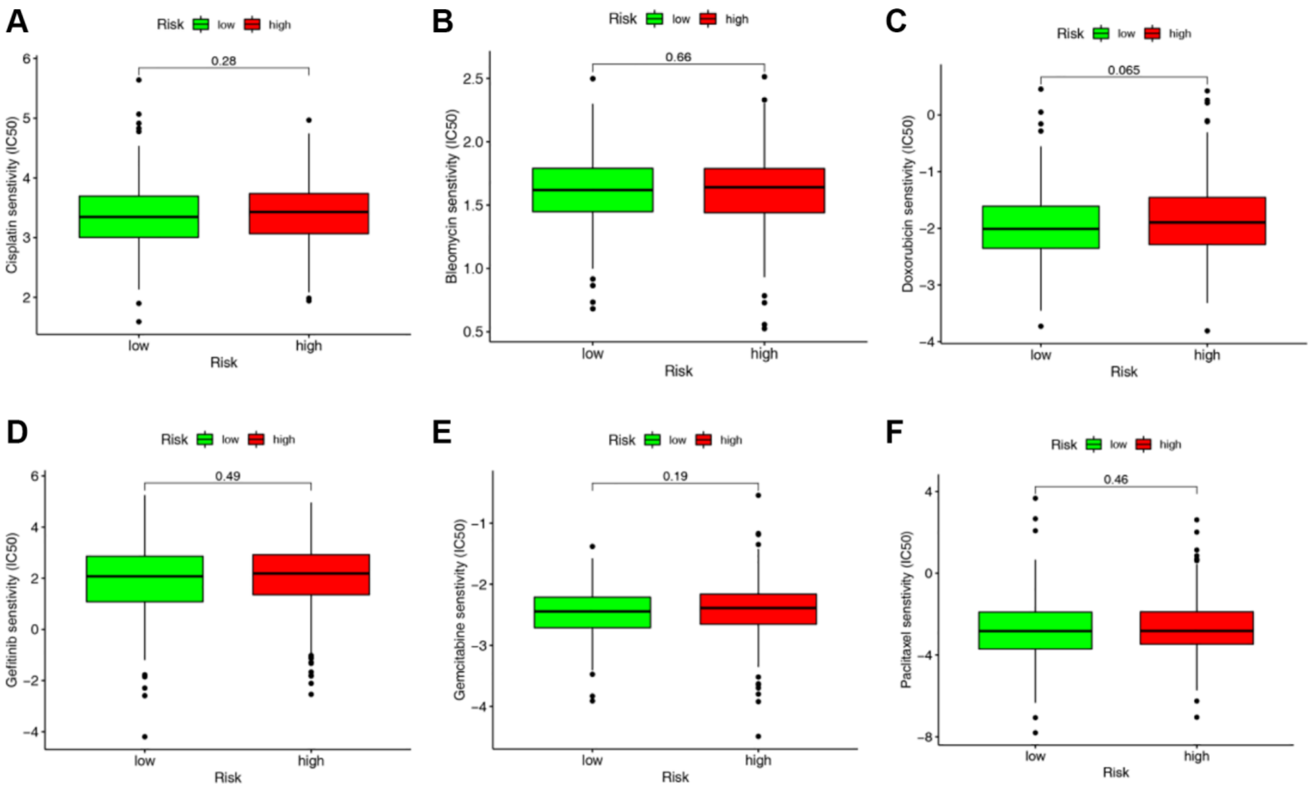

Supplementary Figure 11. Comparisons of the IC50 values of common HNSCC drugs between high-risk and low-risk groups. cisplatin (**A**), Bleomycin (**B**), Doxorubicin (**C**), Gefitinib (**D**), Gemcitabine (**E**), Paclitaxel (**F**).

Supplementary Tables

Please browse Full Text version to see the data of Supplementary Tables 1, 3, 5 and 8.

Supplementary Table 1. 29 Immune gene signatures (gene sets).

Supplementary Table 2. qRT-PCR primer sequences.

CCL2	Forward	GATCTCAGTGCAGAGGCTCG
	Reverse	TCTCCTTGGCCACAATGGTC
CD19	Forward	CTCCCATACCTCCCTGGTCA
	Reverse	GCCCATGACCCACATCTCTC
CTSG	Forward	GAGTCAGACGGAATCGAAACG
	Reverse	CGGAGTGTATCTGTTCCCCTC
RBP5	Forward	CTGGCGTCCCAAATGAAAGA
	Reverse	GAGAGCGGAGATTGGTTGTTCT
ROBO1	Forward	TCCACACAGCAATAGCGAAG
	Reverse	CCTGTAACATGGGCTGGAGT
STC2	Forward	ATGCTACCTCAAGCACGACC
	Reverse	TCTGCTCACACTGAACC
TNFSFR4	Forward	ATGGAAGGGGAAGGGGTTCAACC
	Reverse	TCACAGTGGTACTTGGTTCACAG
ZAP70	Forward	GTTGACTCATCCTCAGAGACGAAT
	Reverse	AGGTTATCGCGCTTCAGGAA
CD247	Forward	GGCACAGTTGCCGATTACAGA
	Reverse	CTGCTGAACTTCACTCTCAGG

Supplementary Table 3. ssGSEA analysis of 29 immune gene signatures in HNSCC samples.

Supplementary Table 4. Clinicopathological features of the training set and the test set.

Total	train set (TCGA-HNSC)	test set (GSE65858)	*p* value (Chi-Square Test)
	499	270	
**Age**			
≤65	324	86	**<0.001**
>65	175	184	
**Gender**			
Female	133	47	**0.004**
Male	366	223	
**T stage**			
T0-1	46	35	0.728
T2	131	80	
T3	96	58	
T4	171	97	
Unknown	55	0	

**N stage**			
N0	170	94	
N1	65	32	**0.015**
N2-3	171	144	
Nx	93	0	
**M stage**			
M0	185	263	
M1	1	7	0.201
Mx	313	0	
**NCCN stage**			
I	25	18	
II	69	37	
III	78	37	0.302
IV	259	178	
Unknown	68	0	
**Grade**			
G1	61	NA	
G2	298	NA	
G3	119	NA	NA
G4	2	NA	
Unknown	19	NA	

Supplementary Table 5. Construction of the immune cell funtion (ICF) gene signature in HNSCC.

Supplementary Table 6. Comparisons of somatic variances between high- and low- risk groups.

Gene	Low-mutation (%)	Low-wild (%)	High-mutation (%)	High-wild (%)	*P*-value (chi-square test)
TP53	134 (54.7)	111 (45.3)	173 (70)	74 (30)	<0.001
TNN	81 (33.1)	164 (66.9)	91 (36.8)	156 (63.2)	0.379
FAT1	49 (20)	196 (80)	54 (21.9)	193 (78.1)	0.612
CDKN2A	44 (18)	201 (82)	44 (17.8)	203 (82.2)	0.966
MUC16	42 (17.1)	203 (82.9)	40 (16.2)	207 (83.8)	0.778
CSMD3	42 (17.1)	203 (82.9)	40 (16.2)	207 (83.8)	0.778
PIK3CA	39 (15.9)	206 (84.1)	40 (16.2)	207 (83.8)	0.934
NOTCH1	39 (15.9)	206 (84.1)	37 (15)	210 (85)	0.773
SYNE1	39 (15.9)	206 (84.1)	35 (14.2)	212 (85.8)	0.588
LRP1B	32 (13.1)	213 (86.9)	35 (14.2)	212 (85.8)	0.72
KMT2D	20 (8.2)	225 (91.8)	42 (17)	205 (83)	0.003
PCLO	27 (11)	218 (89)	32 (13)	215 (87)	0.509
NSD1	34 (13.9)	211 (86.1)	20 (8.1)	227 (91.9)	0.04
DNAH5	27 (11)	218 (89)	27 (10.9)	220 (89.1)	0.975
USH2A	29 (11.8)	216 (88.2)	22 (8.9)	225 (91.1)	0.286
FLG	22 (9)	223 (91)	27 (10.9)	220 (89.1)	0.47
CASP8	27 (11)	218 (89)	20 (8.1)	227 (91.9)	0.27

RYR2	20 (8.2)	225 (91.8)	22 (8.9)	225 (91.1)	0.768
PKHD1L1	17 (6.9)	228 (93.1)	25 (10.1)	222 (89.9)	0.207
XIRP2	25 (10.2)	220 (89.8)	15 (6.1)	232 (93.9)	0.094

Table 7. Molecular docking results of the top 10 compounds.

Rank	ZINC ID	Compound name	Binding energy (kcal/mol)
1	ZINC000011679756	Eltrombopag	−8.3
2	ZINC000116473771	none	−8
3	ZINC000012503187	Conivaptan	−7.8
4	ZINC000003784182	Differin	−7.6
5	ZINC000052955754	Ergotamine	−7.5
6	ZINC000003945984	Vexol	−7.5
7	ZINC000003795819	Palonosetron	−7.4
8	ZINC000084668739	Lifitegrast	−7.4
9	ZINC000003875484	Androxy	−7.3
10	ZINC000064033452	Lumacaftor	−7.3

Supplementary Table 8. Genes in the Black Module of WGCNA.
