# Supplementary material for: Characterization of the immune cell function landscape in head and neck squamous carcinoma to assist in prognosis prediction and immunotherapy
Source: Aging (Albany NY). 2023 Nov 10;15(21):12588–617. doi: 10.18632/aging.205201 (PMC10683602; doi:10.18632/aging.205201)
Supplement: Supplementary Figures [file aging-15-205201-s002.pdf]

SUPPLEMENTARY FIGURES

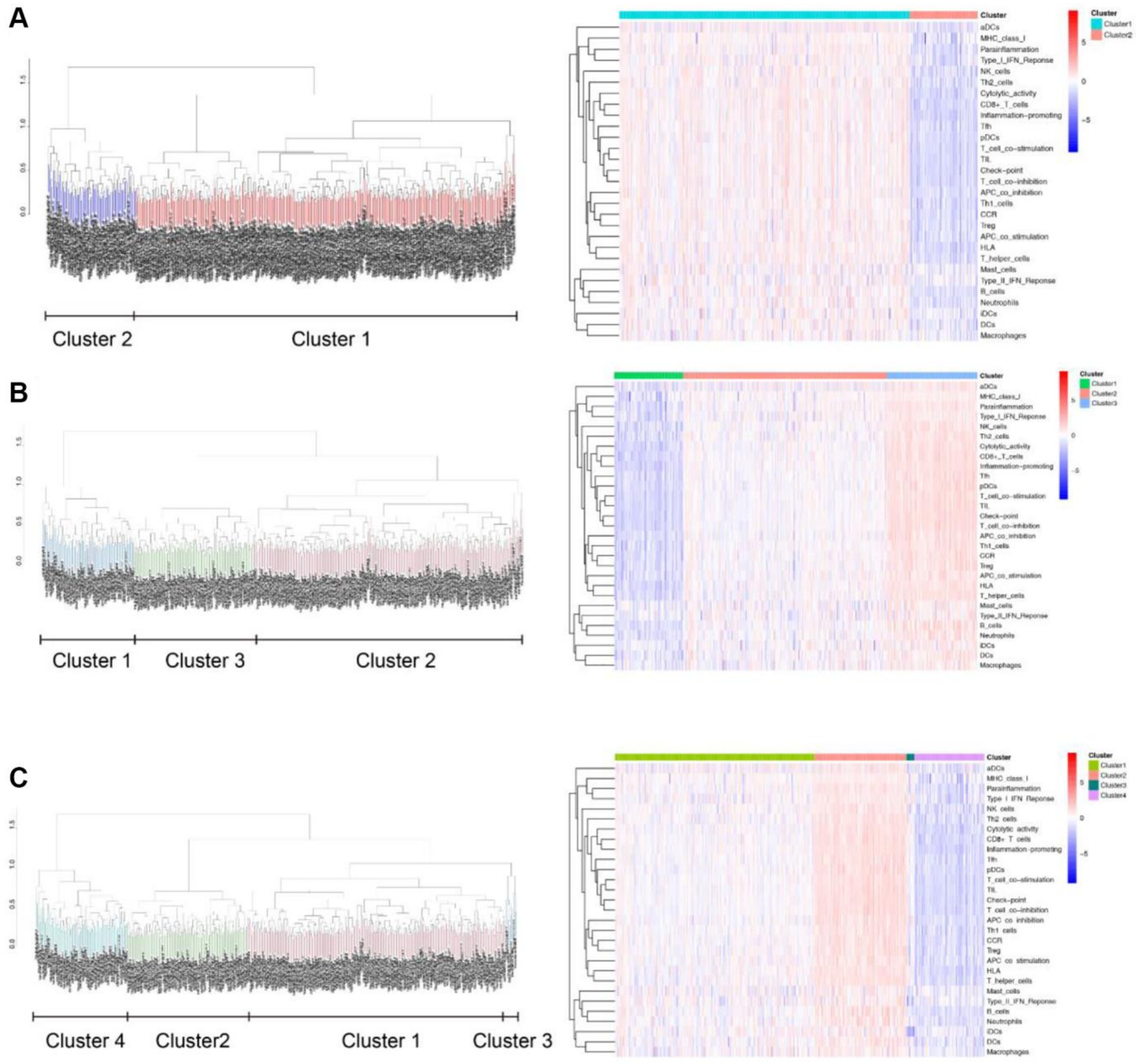

Supplementary Figure 1. Cluster dendrogram (left panel) and the enrichment analysis of 29 functional immune cells (right panel) when TCGA-HNSCC samples were divided into 2 (**A**), 3 (**B**) and 4 (**C**) clusters.

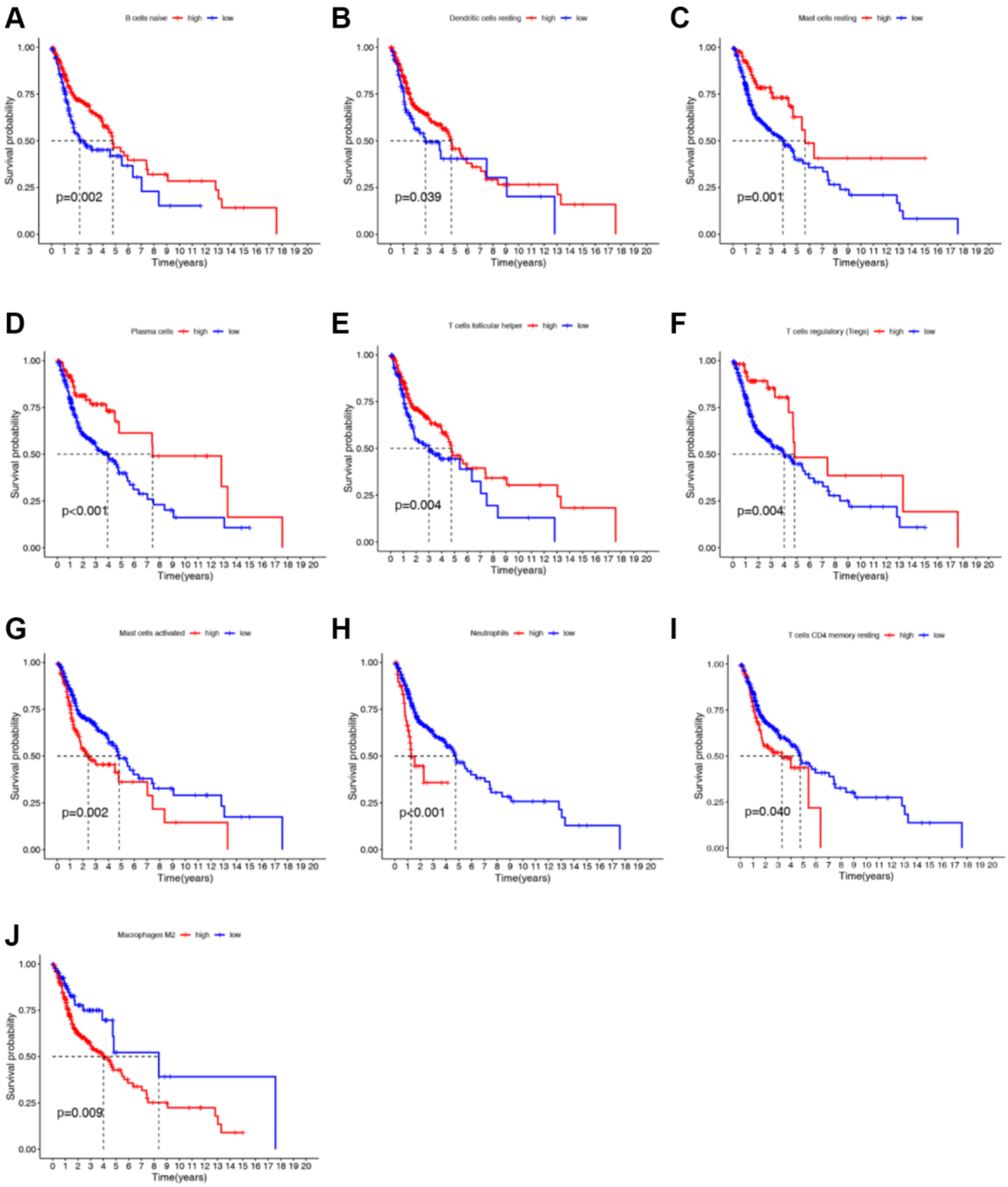

Supplementary Figure 2. Survival analyses based on immune cell infiltration. (**A**) Naïve B cells, (**B**) resting dendritic cells, (**C**) resting mast cells, (**D**) plasma cells, (**E**) T follicular helper cells, (**F**) regulator T cells, (**G**) activated mast cells, (**H**) neutrophils, (**I**) memory resting CD4+ T cells, and (**J**) M2 macrophages.

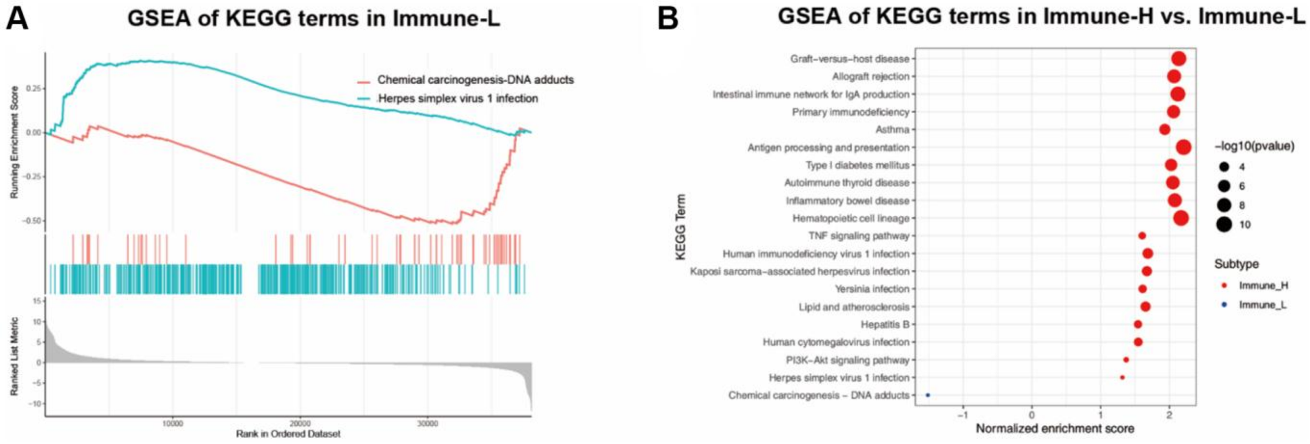

Supplementary Figure 3 Biological processes in different immunophenotypes. (**A**) GSEA shows only the chemical carcinogenesis (DNA adducts) pathway is enriched in the Immune-L subtype. (**B**) The bubble plot shows the enrichment result of KEGG terms in Immune-H and Immune-L subtypes.

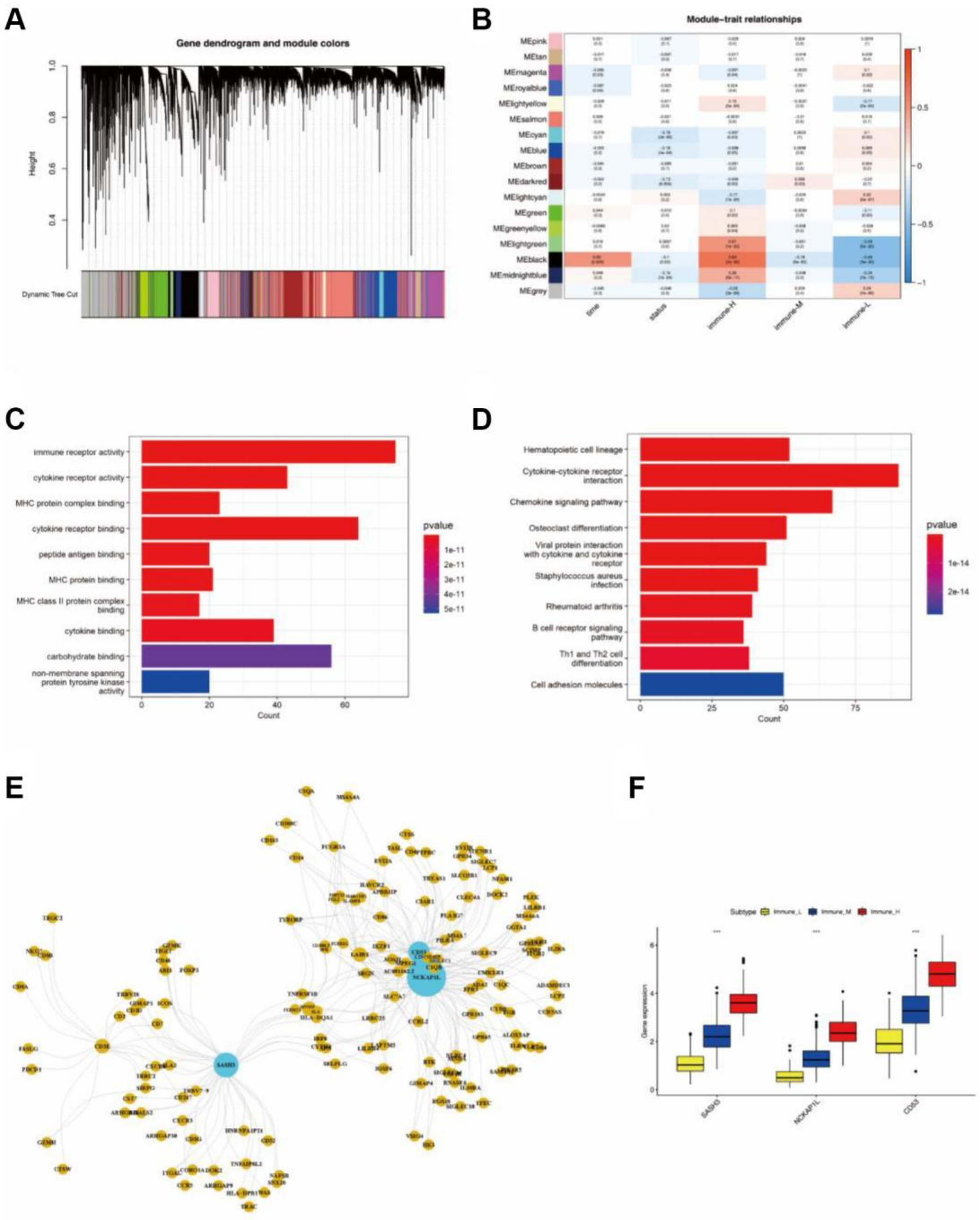

Supplementary Figure 4. Identification of the immunophenotype-related gene module by WGCNA. (**A**) Cluster dendrogram of gene co-expression modules after merged (1-TOM). (**B**) Correlation analysis of gene modules and phenotypes of HNSCC. GO functional enrichment (**C**) and KEGG pathway enrichment (**D**) analyses for genes in the black module of WGCNA. (**E**) The protein-protein interaction network of the hub genes in the black module. (**F**) The expression levels of SAHS3, CD53 and NCKAP1L in the three immunophenotypes.

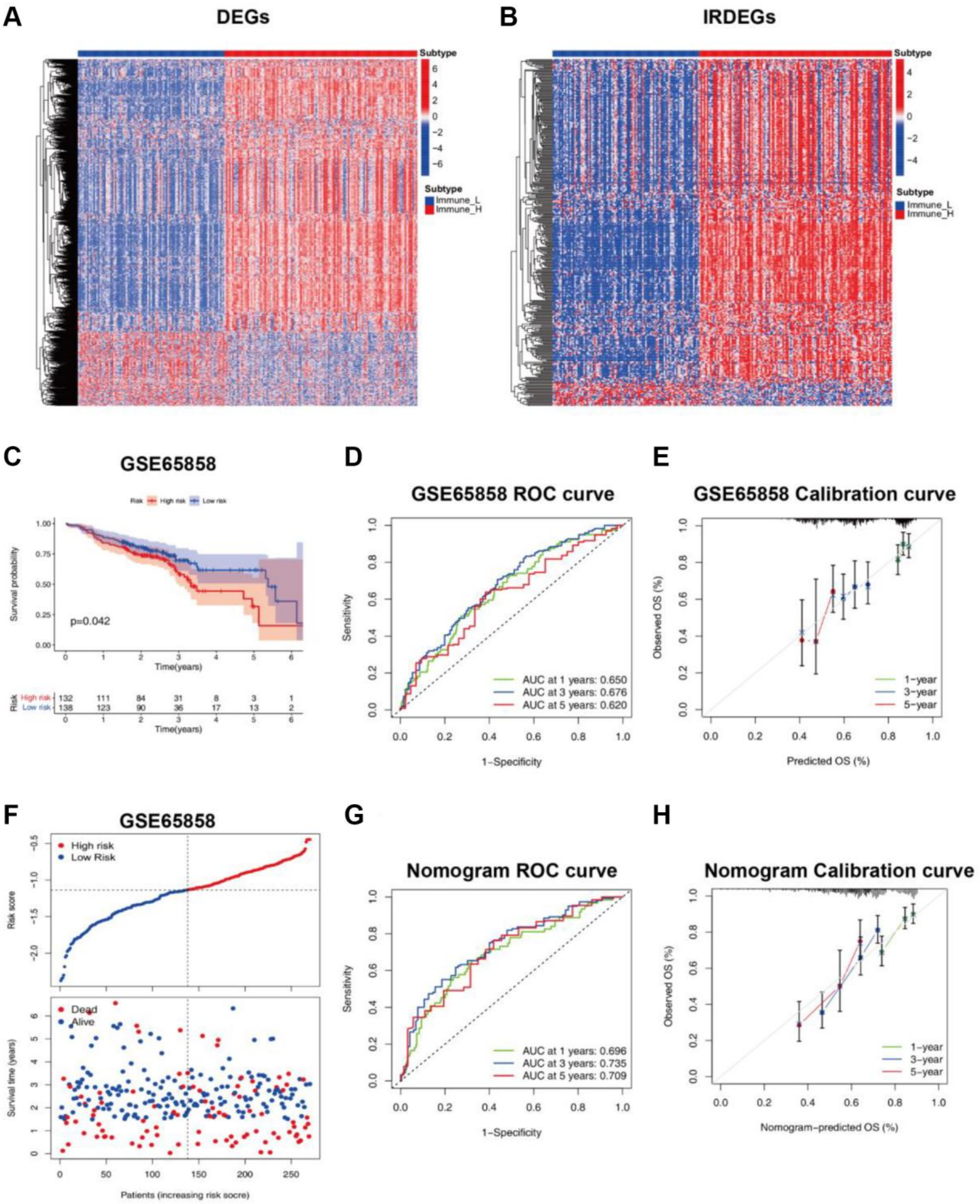

Supplementary Figure 5. Construction and validation of the ICF gene signature. Heatmap of DEGs (**A**) and IRDEGs (**B**) between the Immune-H and Immune-L subtypes. (**C**) Comparisons of overall survival between high- and low-risk groups in the test set. The ROC curve (**D**) and calibration curve (**E**) of the ICF score for predicting 1-year, 3-year and 5-year survival in the test set. (**F**) Correspondence between risk scores and survival in the test set. The ROC curve (**G**) and calibration curve (**H**) of the nomogram for predicting overall survival at 1-year, 3-year and 5-year.

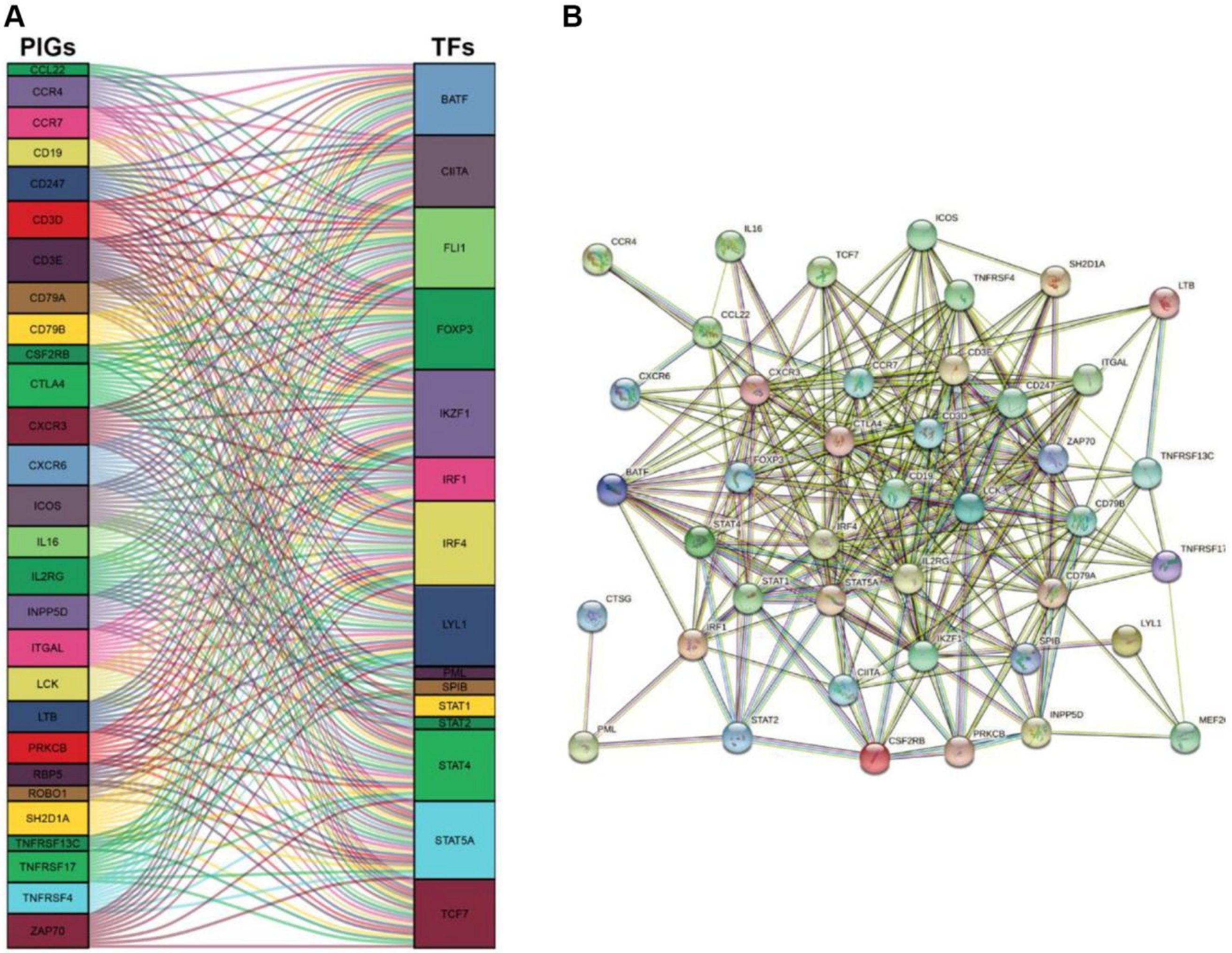

Supplementary Figure 6. Analysis of candidate transcription factors (TFs) for prognosis-related immune genes (PIGs). (**A**) The alluvial diagram shows the TFs co-expressed with PIGs, and the lines indicate the co-expressed relationship between the two. (**B**) Protein-protein interaction network of PIGs and TFs.

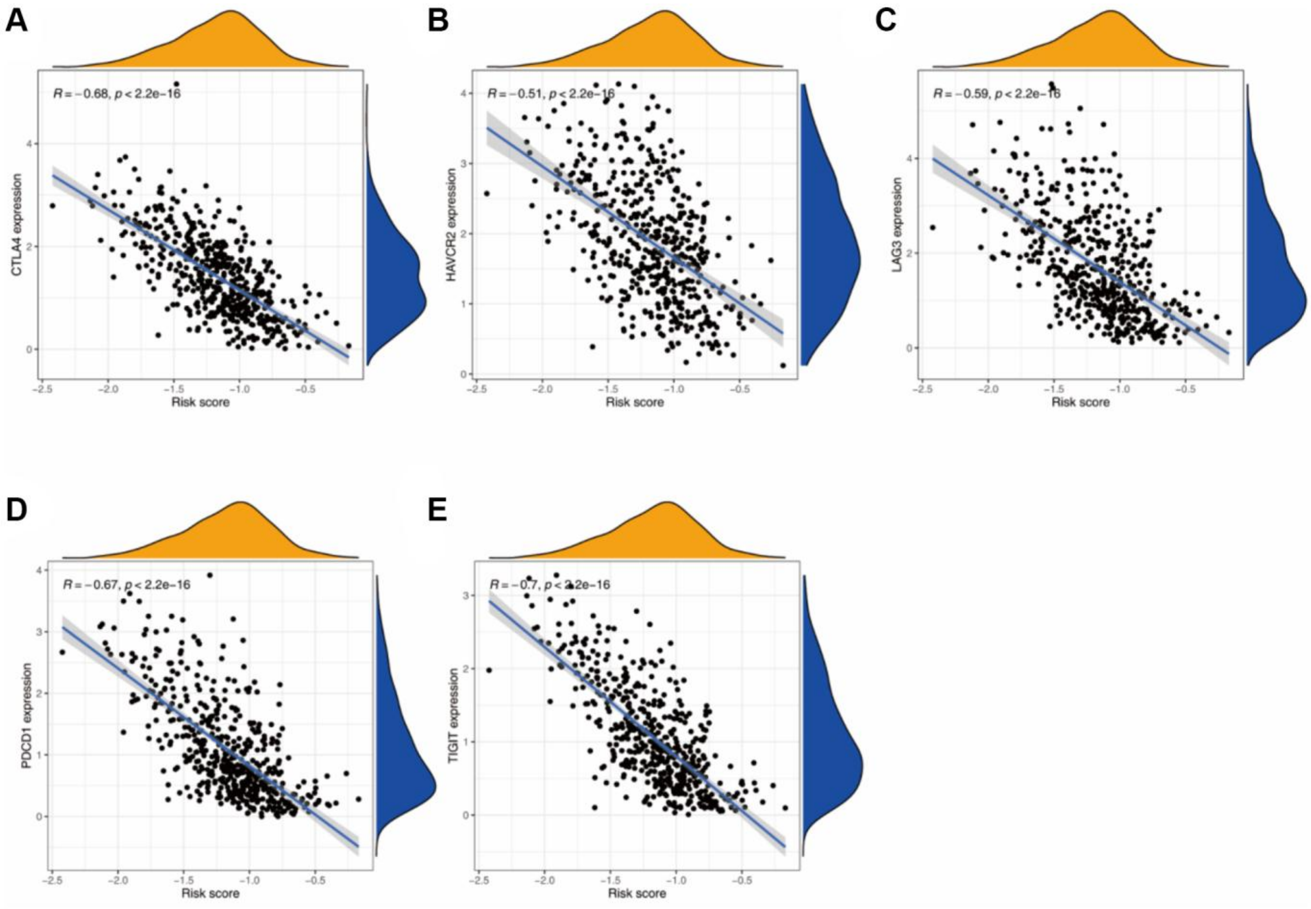

Supplementary Figure 7. The correlations between immune checkpoints and ICF scores in HNSCC. CTLA4 (**A**), HAVCR2 (**B**), LAG3 (**C**), PDCD1 (**D**), and TIGIT (**E**).

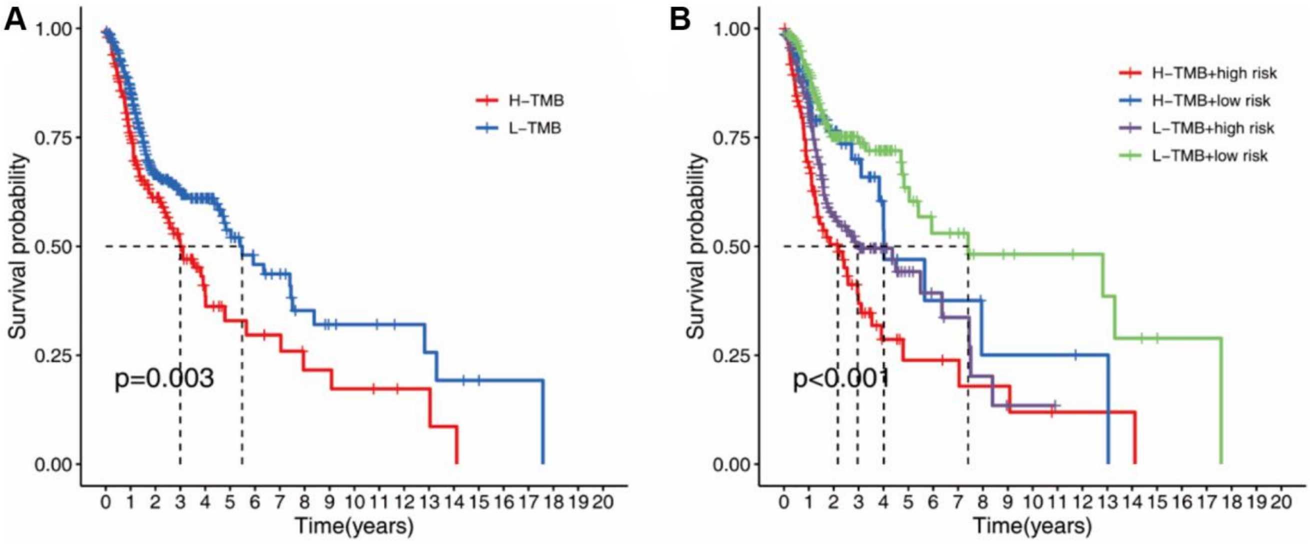

Supplementary Figure 8. Survival analysis for HNSCC patients stratified by TMB and ICF risk score. (**A**) Kaplan-Meier survival analysis for HNSCC patients with different TMB. (**B**) Kaplan-Meier survival analysis for patients according to TMB and ICF score stratifications.

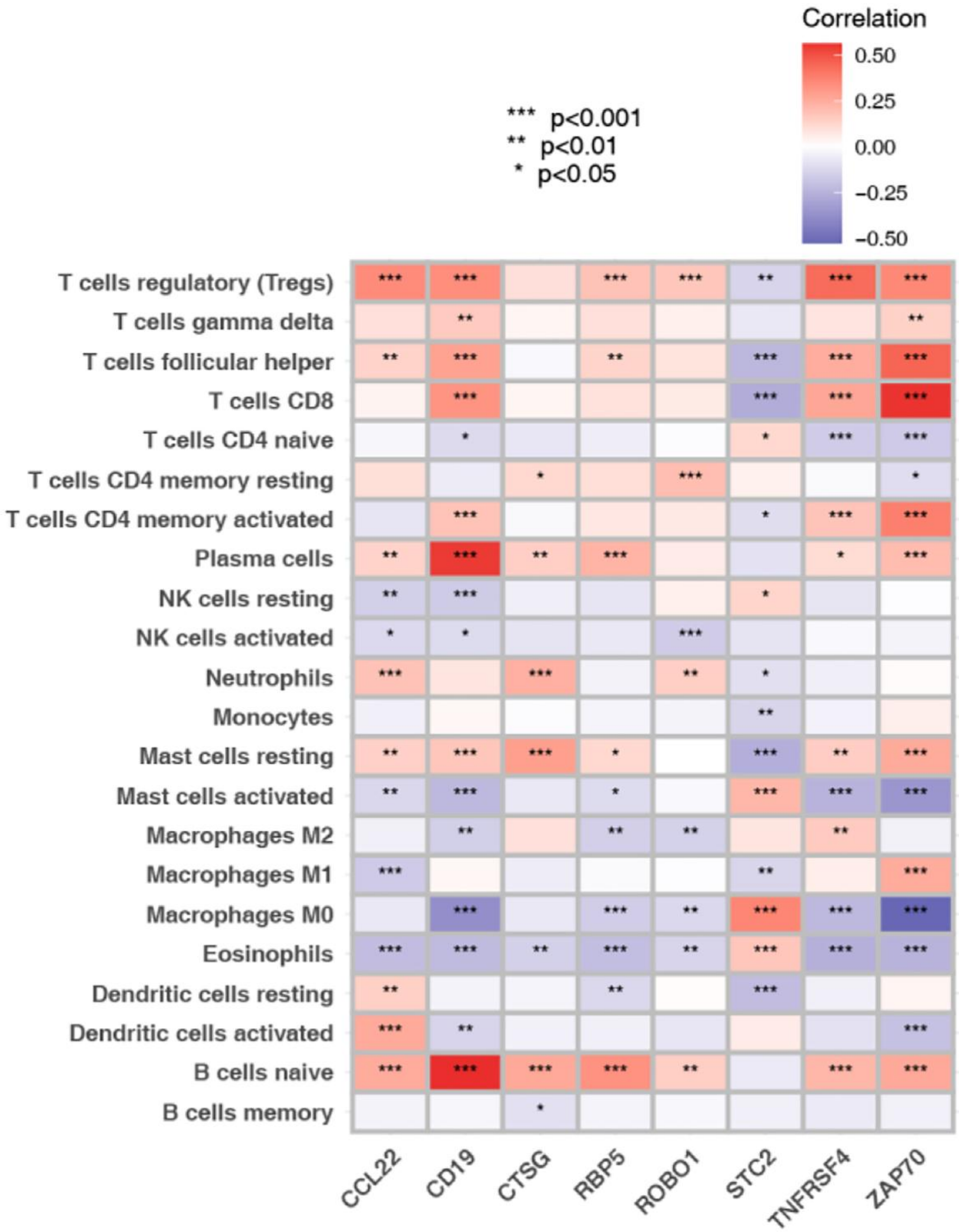

Supplementary Figure 9. Correlation analyses between the model genes and immune infiltrating cells.

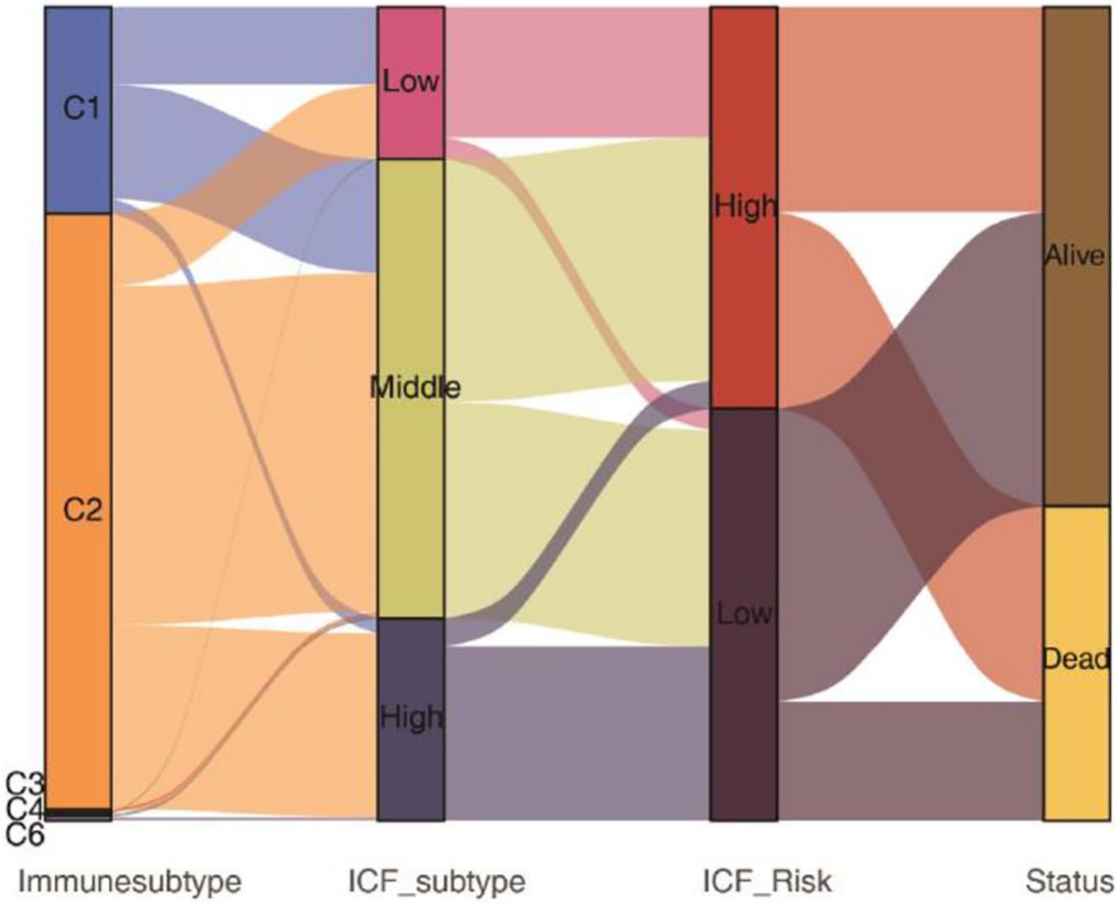

Supplementary Figure 10. The alluvial diagram shows the correspondence of pan-cancer immune subtypes, ICF subtypes, ICF risk groups and the survival status in HNSCC.

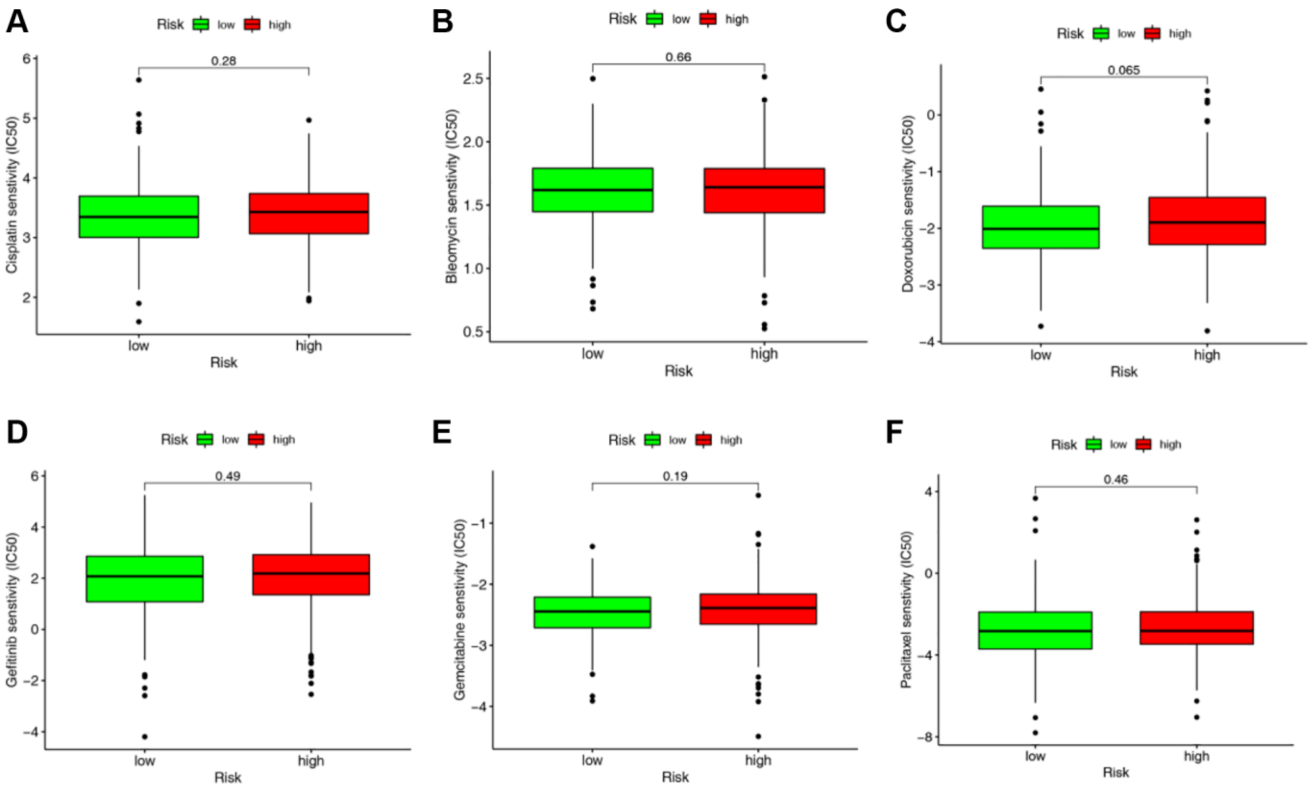

Supplementary Figure 11. Comparisons of the IC50 values of common HNSCC drugs between high-risk and low-risk groups. Cisplatin (**A**), Bleomycin (**B**), Doxorubicin (**C**), Gefitinib (**D**), Gemcitabine (**E**), Paclitaxel (**F**).
