# Supplementary material for: Characterization of the immune cell function landscape in head and neck squamous carcinoma to assist in prognosis prediction and immunotherapy
Source: Aging (Albany NY). 2023 Nov 10;15(21):12588–617. doi: 10.18632/aging.205201 (PMC10683602; doi:10.18632/aging.205201)
Supplement: Supplementary Tables 2, 4 and 6-7 [file aging-15-205201-s003.pdf]

SUPPLEMENTARY TABLES

Supplementary Table 2. qRT-PCR primer sequences.

CCL2	Forward	GATCTCAGTGCAGAGGCTCG
	Reverse	TCTCCTTGGCCACAATGGTC
CD19	Forward	CTCCCATACCTCCCTGGTCA
	Reverse	GCCCATGACCCACATCTCTC
CTSG	Forward	GAGTCAGACGGAATCGAAACG
	Reverse	CGGAGTGTATCTGTTCCCCTC
RBP5	Forward	CTGGCGTCCCAAATGAAAGA
	Reverse	GAGAGCGGAGATTGGTTGTTCT
ROBO1	Forward	TCCACACAGCAATAGCGAAG
	Reverse	CCTGTAACATGGGCTGGAGT
STC2	Forward	ATGCTACCTCAAGCACGACC
	Reverse	TCTGCTCACACTGAACC
TNFSFR4	Forward	ATGGAAGGGGAAGGGGTTCAACC
	Reverse	TCACAGTGGTACTTGGTTCACAG
ZAP70	Forward	GTTGACTCATCCTCAGAGACGAAT
	Reverse	AGGTTATCGCGCTTCAGGAA
CD247	Forward	GGCACAGTTGCCGATTACAGA
	Reverse	CTGCTGAACTTCACTCTCAGG

Supplementary Table 4. Clinicopathological features of the training set and the test set.

**Total**	**train set (TCGA-HNSC)**	**test set (GSE65858)**	***P*-value (Chi-Square Test)**
	499	270	
**Age**			
≤65	324	86	**<0.001**
>65	175	184	
**Gender**			
Female	133	47	**0.004**
Male	366	223	
**T stage**			
T0-1	46	35	0.728
T2	131	80	
T3	96	58	
T4	171	97	
Unknown	55	0	
**N stage**			
N0	170	94	**0.015**
N1	65	32	
N2-3	171	144	
Nx	93	0	
**M stage**			
M0	185	263	0.201

M1	1	7	
Mx	313	0	
**NCCN stage**			
I	25	18	
II	69	37	0.302
III	78	37	
IV	259	178	
Unknown	68	0	
**Grade**			
G1	61	NA	
G2	298	NA	NA
G3	119	NA	
G4	2	NA	
Unknown	19	NA	

Supplementary Table 6. Comparisons of somatic variances between high- and low- risk groups.

Gene	Low-mutation (%)	Low-wild (%)	High-mutation (%)	High-wild (%)	*P*-value (chi-square test)
TP53	134 (54.7)	111 (45.3)	173 (70)	74 (30)	<0.001
TNN	81 (33.1)	164 (66.9)	91 (36.8)	156 (63.2)	0.379
FAT1	49 (20)	196 (80)	54 (21.9)	193 (78.1)	0.612
CDKN2A	44 (18)	201 (82)	44 (17.8)	203 (82.2)	0.966
MUC16	42 (17.1)	203 (82.9)	40 (16.2)	207 (83.8)	0.778
CSMD3	42 (17.1)	203 (82.9)	40 (16.2)	207 (83.8)	0.778
PIK3CA	39 (15.9)	206 (84.1)	40 (16.2)	207 (83.8)	0.934
NOTCH1	39 (15.9)	206 (84.1)	37 (15)	210 (85)	0.773
SYNE1	39 (15.9)	206 (84.1)	35 (14.2)	212 (85.8)	0.588
LRP1B	32 (13.1)	213 (86.9)	35 (14.2)	212 (85.8)	0.72
KMT2D	20 (8.2)	225 (91.8)	42 (17)	205 (83)	0.003
PCLO	27 (11)	218 (89)	32 (13)	215 (87)	0.509
NSD1	34 (13.9)	211 (86.1)	20 (8.1)	227 (91.9)	0.04
DNAH5	27 (11)	218 (89)	27 (10.9)	220 (89.1)	0.975
USH2A	29 (11.8)	216 (88.2)	22 (8.9)	225 (91.1)	0.286
FLG	22 (9)	223 (91)	27 (10.9)	220 (89.1)	0.47
CASP8	27 (11)	218 (89)	20 (8.1)	227 (91.9)	0.27
RYR2	20 (8.2)	225 (91.8)	22 (8.9)	225 (91.1)	0.768
PKHD1L1	17 (6.9)	228 (93.1)	25 (10.1)	222 (89.9)	0.207
XIRP2	25 (10.2)	220 (89.8)	15 (6.1)	232 (93.9)	0.094

Supplementary Table 7. Molecular docking results of the top 10 compounds.

Rank	ZINC ID	Compound name	Binding energy (kcal/mol)
1	ZINC000011679756	Eltrombopag	−8.3
2	ZINC000116473771	none	−8
3	ZINC000012503187	Conivaptan	−7.8

4	ZINC000003784182	Differin	−7.6
5	ZINC000052955754	Ergotamine	−7.5
6	ZINC000003945984	Vexol	−7.5
7	ZINC000003795819	Palonosetron	−7.4
8	ZINC000084668739	Lifitegrast	−7.4
9	ZINC000003875484	Androxy	−7.3
10	ZINC000064033452	Lumacaftor	−7.3
